# The origin of a derived superkingdom: how a gram-positive bacterium crossed the desert to become an archaeon

**DOI:** 10.1186/1745-6150-6-16

**Published:** 2011-02-28

**Authors:** Ruben E Valas, Philip E Bourne

**Affiliations:** 1 Bioinformatics Program, University of California, San Diego, 9500 Gilman Drive, La Jolla, CA 92093, USA; 2 Skaggs School of Pharmacy and Pharmaceutical Sciences, University of California, San Diego, 9500 Gilman Drive, La Jolla, CA 92093, USA

## Abstract

**Background:**

The tree of life is usually rooted between archaea and bacteria. We have previously presented three arguments that support placing the root of the tree of life in bacteria. The data have been dismissed because those who support the canonical rooting between the prokaryotic superkingdoms cannot imagine how the vast divide between the prokaryotic superkingdoms could be crossed.

**Results:**

We review the evidence that archaea are derived, as well as their biggest differences with bacteria. We argue that using novel data the gap between the superkingdoms is not insurmountable. We consider whether archaea are holophyletic or paraphyletic; essential to understanding their origin. Finally, we review several hypotheses on the origins of archaea and, where possible, evaluate each hypothesis using bioinformatics tools. As a result we argue for a firmicute ancestry for archaea over proposals for an actinobacterial ancestry.

**Conclusion:**

We believe a synthesis of the hypotheses of Lake, Gupta, and Cavalier-Smith is possible where a combination of antibiotic warfare and viral endosymbiosis in the bacilli led to dramatic changes in a bacterium that resulted in the birth of archaea and eukaryotes.

**Reviewers:**

*This article was *reviewed by Patrick Forterre, Eugene Koonin, and Gáspár Jékely

## Background

Archaea were first discovered because of a distinct sequence signature in their ribosomal RNA [1]. This remains one of the strongest signals found anywhere in the phylogenetic tree. It was truly a revolution in thought when the world realized there were two distinct types of prokaryotes. Besides placement on sequence trees, there are three major areas where archaea and bacteria differ greatly. First, the structures of archaeal and bacterial ribosomes each have many unique proteins [2]. Second, archaeal membranes are composed of glycerol-ether lipids, while bacterial membranes are composed of glycerol-ester lipids [3]. The glycerols have different stereochemistries between the superkingdoms as well. Third, the DNA replication machinery of these two superkingdoms is very different; many key proteins comprising this machinery have a superkingdom specific distribution [4].

These differences as well as the rRNA tree have convinced most scientists that the root of the tree of life must be between the prokaryotic superkingdoms. The proposal that archaea were a different kingdom was originally considered ridiculous because no one could imagine two distinct groups of prokaryotes [5]. In 30 years we went from the prevailing opinion that the archaea were similar enough to bacteria to be just prokaryotes, to the view they are so different they must each be primordial lineages.

Locating the root of the tree of life is a prerequisite for understanding the origin and evolution of life. There are many examples of conclusions that become radically different if one assumes a different rooting of the tree. For example, the proposal that LUCA was acellular relies on a rooting between the archaea and bacteria [6]. Each of the estimates for divergence times of the prokaryotic taxa [7] would change drastically if archaea are not the same age as LUCA.

Several groups have hypothesized that the root of the tree of life lies within bacteria and place archaea as a taxon derived from gram-positive bacteria [8-10]. These hypotheses are often dismissed for two reasons: 1) they do not agree on a single rooting; 2) there is an immense gap between archaea and bacteria in sequence trees and in the systems mentioned above. We addressed the differences between these alternative rooting options in [11] and concluded that it is possible for them to converge on a single root in the gram-negative bacteria. The point of this work is to address the objection to rooting archaea within the gram-positives.

This work is a synthesis of many creative ideas that came before us; as a result, much of what we say here has been said in some form before by others. However, the arrangement of the pieces is, we believe, novel and sheds light on the strengths and weaknesses of the various rootings of the tree of life. First, we discuss the ideas in their original form and consider what we see as the strengths and weaknesses of each. We take the stance that closing the debate prematurely deprives one of the ability to the see the many strengths of each of these hypotheses and the large common ground between them. We then offer novel data that helps refine some of these ideas and show the potential for testing them further.

Radhey Gupta and colleagues created a detailed tree of life using rarely fixed indels (insertion-deletions) in prokaryotic groups [9]. He concluded that the root of the tree of life is within the gram-positive bacteria, and he places archaea as derived from firmicutes. The major driving force in his scenario is antibiotic warfare. He argues the differences between archaea and bacteria coincide with many of the targets of antibiotics produced by gram-positive bacteria. We will review recent work that demonstrates many antibiotic binding sites have dramatically different affinities in the superkingdoms. The strength of Gupta's phylogeny rests on the fact that many of the branch orders are supported by several independent indels. However, there are several points that concern us about Gupta's hypothesis. First, we disagree with his polarization of Hsp70 which is used to justify the root of the tree of life [11]. But the focus of the present paper is the origin of archaea, so that debate is probably better left to our other work[11]. The transition between gram-positives and archaea must have been a drastic event to be confronted in any hypothesis that roots the tree of life in bacteria. Antibiotic warfare is a powerful evolutionary force, but in Gupta's hypothesis there seems to be a special battle that resulted in archaea. He does not explain why antibiotic warfare only gave rise to one other prokaryotic superkingdom. Should not one expect there to be several different modified ribosomes in response to antibiotic pressure? We will invoke antibiotic warfare as a major driver in the origin of archaea, but we feel our scenario better sets the stage for why this was a unique event. Antibiotic warfare on its own is not enough to account for the vast differences between the prokaryotic superkingdoms, but it certainly was important.

James Lake and colleagues has also constructed a detailed tree of life using indels [10]. His group has focused more on indels that can be polarized using paralogous out-groups. The strength of Lake et al.'s method is that it provides evidence for derived and ancestral groups, which we feel is essential for understanding evolutionary histories. The polarizations are largely independent. This allows one to refine the tree because a flawed polarization will only affect one part of the tree. Like Gupta, his group roots archaea within the firmicutes and provides several independent reasons why this makes sense [12]. Lake has also proposed that eukaryotes had a crenarchaeal (eocyte) origin based on a shared indel in EF-1 and similarities in their ribosomal structure [13,14]. We find arguments like this appealing as it is a synthesis of both sequence and structural data. We discuss the strengths and weaknesses of that particular hypothesis at length below.

The weakness of the indel method in general is the difficulty in properly aligning paralogs as we argued in [11]. Fortunately, polarizations are mostly independent; so changing a polarization does not invalidate the whole tree, it just refines it. We argue that the refined version of Lake's tree is completely consistent with Cavalier-Smith's [11]. There are very few universal paralogs, so this method certainly needs to be supplemented with other data sources.

Cavalier-Smith has discussed the relationship and origin of the superkingdoms at length [8,15,16]. The major difference between his hypothesis and that of Gupta and Lake is the placement of the root in the gram-negative bacteria. He also roots archaea within or next to the actinobacteria. Cavalier-Smith constructed his tree by polarizing multiple types of data including indels, membrane structure, and quaternary structure. Again, if any one of these polarizations is brought into question it does not weaken the remainder. Cavalier-Smith included unique supporting discussions from the prokaryotic fossil. His analysis concludes that there is no fossil to indicate archaea are older than eukaryotes, despite much evidence that bacteria are older than eukaryotes. That said, there are several aspects of Cavalier-Smith's tree that still do not sit well with us. His hypothesis relies on the assumption that archaea are holophyletic (eukaryotes are their sisters, not their descendents). He provides some justification for this, but we will discuss below why we believe this is not a completely safe assumption at this time. Cavalier-Smith's rooting of the neomura (his term for archaea, eukaryotes and their last common ancestor (LAECA)) is in the actinobacteria. He cites traits shared between the eukaryotes and actinobacteria to support this hypothesis, but they are only relevant if archaea are holophyletic. We provide an alternative interpretation of this distribution by invoking an actinobacterial endosymbiont near the root of eukaryotes. Cavalier-Smith argues thermophily was the major force that lead to the neomuran revolution. We feel this argument falls short for the same reason as Gupta's; it just does not seem to be a unique enough selective pressure to create a novel superkingdom. Cavalier-Smith prefers the labels archaebacteria and eubacteria because he feels the labels archaea and bacteria over emphasize the difference between these superkingdoms. We disagree, these superkingdoms are fundamentally different. Despite that, we still believe archaea evolved from within bacteria.

None of these scenarios adequately addresses the origin of the DNA replication machinery shared between archaea and eukaryotes. Therefore we invoke the ideas of Patrick Forterre, who has proposed that cells received the ability to replicate DNA from viruses. He proposes this occurred three times; each event resulting in the birth of a superkingdom [17,18]. The amazing variation in DNA replication machinery found throughout the virosphere supports this idea. All extant cells uses double stranded DNA, but viruses can have many other forms of genetic material (reviewed in [19]). The plasticity of replication in the virus world certainly could lead to innovations of great importance in the cellular world.

There are two weaknesses to this view in our opinion. First, it is DNA centric so it necessarily neglects the many other important differences between the superkingdoms. Second, it is firmly placed within the framework of the classical rRNA tree. Forterre even assumes eukaryotes are a primordial lineage, as a consequence of taking the sequence tree too literally. We will demonstrate that this view is also highly informative if archaea are derived from bacteria. It has also been noted that other extra chromosomal elements could play key roles in the evolution of the different DNA replication systems [20], but that discussion is also firmly grounded in the canonical rooting.

Taking all these viewpoints together, it would seem an uphill battle to argue that archaea are a derived superkingdom. One needs to provide compelling evidence archaea are derived, so we will review our data that supports that view. Any hypothesis that addresses how a bacterium could become an archaeon would have to explain dramatic changes in membranes, DNA replication, and ribosomes. We will demonstrate that the ribosome can have great plasticity under certain circumstances. It has been previously argued that the firmicutes have many of the enzymes needed to make archaeal membranes [21]. We will invoke viral endosymbiosis to explain the differences in DNA replication. For the reasons discussed below the hypothesis must work if archaea are paraphyletic or holophyletic. Finally, it must also address the rarity of the event that lead to this revolution. If a hypothesis could do all of these things, it would make a compelling argument for the origin of archaea.

## Results

### Three reasons why archaea are derived

Several large indels are shared between archaea and gram-positive bacteria, and both groups only have one membrane [9]. Thus, if there is a direct relationship between the gram-positives and archaea the root is either between them, or one is derived from the other. Every piece of evidence that is polarizable implies archaea are derived from bacteria. Arguments that archaea and bacteria are so different that they both evolved from LUCA sidesteps directionality altogether. The only recent work that explicitly roots the tree in archaea is that of Wong et al. [22]. Many of their arguments are based on assumptions about the nature of LUCA and assumptions of what a primitive state would look like. None of their arguments are true polarizations. To the best of our knowledge there is no single polarized argument for an archaeal rooting that is on par with the three we shall discuss that place archaea as derived.

The first of these arguments is the proteasome. Proteasomes are self compartmentalized atp-dependent proteases that are found in varying degrees of complexity across the tree of life. All archaea contain a 20S proteasome which is composed of 28 subunits and is encoded by at least two genes that are clearly homologs. Therefore the 20S proteasome must be the result of duplication. Cavalier-Smith has argued that the simpler bacterial homolog HslV (heat shock locus v) could be duplicated to generate a 20S proteasome [8,16]. Loss of a subunit in the 20S proteasome would result in an open proteasome with no ATPase. Such a protein would lose the essential function of controlled degradation found in proteasomes, and does not make sense as an intermediate. It is more likely that the 20S proteasome is derived from a simpler structure. Cavalier-Smith excludes the root from archaea because all archaea contain a clearly derived protein.

**Figure 1:**
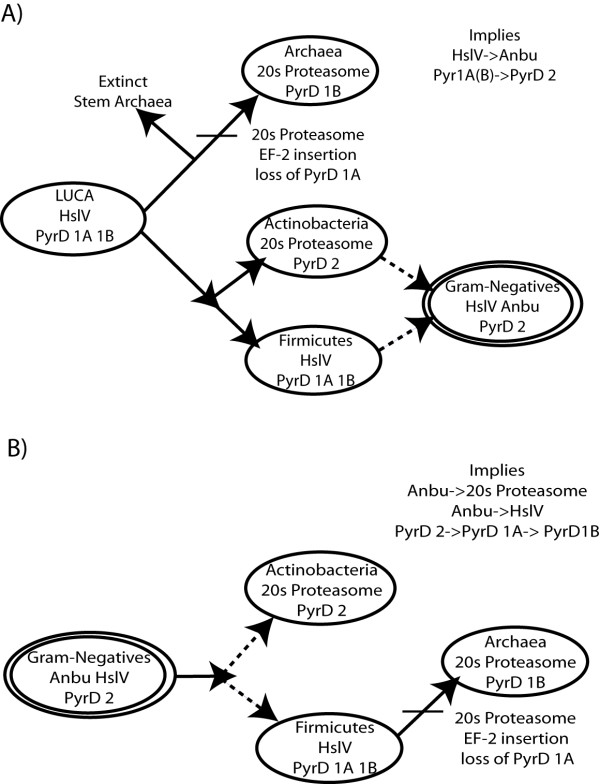
**Two scenarios for interpreting the three polarizations**. A) Under the canonical rooting proteasome evolution would require several selective sweeps and large-scale loss. The monomer PyrD B would have evolved from one of the more complex quaternary structures, and the derived insert in EF-2 would occur after LACA. B) Under the gram-negative rooting, Anbu could be ancestral to both HslV and the 20S proteasome. PyrD could evolve via stepwise increases in structural complexity, and there is no need to invoke extinct stem archaea to explain the EF_G insert. We believe these transitions argue for a gram-negative rooting.

However, there is a counter argument to that proposal; LUCA had HslV and LACA (last archaeal common ancestor) is the point in the tree where HslV evolves into the 20S proteasome (Figure 1A). This would still exclude the root from the crown archaea, but it still allows for the possibility that the root is between the extinct stems of archaea and bacteria. Excluding the root from archaea will never be enough because one can always invoke stem lineages that show up before the derived trait. This would imply the 20S proteasome present in actinobacteria is probably the result of a horizontal transfer from archaea. However, we have observed that the two proteasome genes are often in the same operon in actinobacteria, but rarely together in archaea. This weakly polarizes the direction of the horizontal transfer to the archaea.

However, there is stronger evidence that narrows the root to within the bacteria. Our own work argues that the Anbu proteasome (or peptidase according to [23]) is more likely than HslV to be the 20S proteasome's direct ancestor based on both sequence data and structure predictions [24]. This argument is much stronger than Cavalier-Smith's because HslV is widespread in the gram-positives but Anbu appears to be missing in them altogether (Figure 1B). If the divide between archaea and bacteria is the earliest split in the tree, and our hypothesis on proteasome evolution is correct, then LUCA must have had Anbu. This would mean that all extant gram-positives need to have lost Anbu while the gram-negatives (that must be derived from gram-positives in this scenario) somehow retained Anbu. One would have to invoke a selective sweep of the 20S proteasome in archaea, and of HslV in the gram-positives. It is plausible that the 20S proteasome outcompeted Anbu or HslV since they are almost never found in the same genome. However, Anbu and HslV are found together in many genomes, which is evidence neither totally displaces the other in terms of function. Our arguments about Anbu are based on structure prediction, but a crystal structure could experimentally verify those predictions. If we are correct it may be the smoking gun for a gram-negative rooting, but even without that there is ample evidence to support Cavalier-Smith's position. Even if HslV is the direct ancestor of the 20S proteasome the root can still be excluded from all extant archaeal lineages.

The recent analysis of proteins occur in Anbu's operon [23] presented evidence we are wrong in labeling Anbu a proteasome because it lacks an associated ATP-dependent protein required for unfolding substrates. HslV and the 20S proteasome clearly have associated ATPases dedicated to unfolding substrates. Therefore the transition to both of them is easier from Anbu as no ATPase would have to be lost. The origin of HslV and the 20S proteasome would both involve the recruitment of distinct ATPases subunits. Therefore we think this new work strengthens our hypothesis that Anbu is ancestral to the 20S proteasome because no intermediate would ever lose the regulatory ATPase. If our hypothesis is correct, proteasomes would be polyphyletic if they are defined by the presence of the ATPase subunit as suggested in [23].

The indel in EF-2 shared between archaea and eukaryotes has been polarized using EF-Tu as an outgroup [25]. Our alignment free analysis of this indel agrees with the authors' conclusions despite there being a sequence artifact in their original alignment [11]. This polarization robustly excludes the root from within archaea, but does not narrow it to within bacteria.

In that analysis we also presented a novel structure-based argument for polarizing archaea. The quaternary structure of PyrD 1B is a heterotetramer across the firmicutes and archaea. We argue that the heterotetramer is probably derived from the homodimer PyrD 1A based on the presence of a conserved interface. The monomeric and homodimeric versions are present in the Gram-negatives and Actinobacteria. PyrD 1B is found across a gram-positive group and archaea, so it would have to be present in their last common ancestor, which is LUCA under the canonical rooting. This could be explained by the presence of both PyrD 1A and 1B in LUCA. But that scenario would require PyrD 1A to be lost in every archaea and some firmicutes, and for there to be a reversion to the monomeric form, PyrD, across the gram-negatives and actinobacteria. PyrD 1B is probably derived, so it follows that archaea, firmicutes, and their last common ancestor are also derived.

The polarization of the indel in EF-2 excludes the root from the extant archaea. Our novel polarizations of Anbu and PyrD argue the root is within bacteria. If these arguments only excluded the root from all extant archaea one is left wondering why all archaea that are not clearly derived went extinct. The combination of all three arguments strongly supports the bacterial rooting of the tree. If archaea are derived, there must be some way of reconciling the major differences between them and bacteria.

### Ribosomal revolutions are historical fact

Archaea cluster separately on phylogenetic trees based on ribosomal RNA [1]. This split has remained robust in many trees derived since then. We will discuss three scenarios that can explain this. The first scenario is that the ribosomal sequences are pretty good molecular clocks. The great splits seen in the tree reflects this most ancient divide in cellular life and is in accordance with the canonical rooting.

The second scenario also does not contradict the canonical rooting. It goes as follows. The ribosome in LUCA was incomplete. It did not have all the proteins found in extant archaea and bacteria, only the core that is universal between them. The addition of proteins after the split of the superkingdoms would start a quantum evolutionary event. Some sites would be free to mutate to achieve increased stability, while others would be under evolutionary pressure to maintain a strict structure-function relationship. The rate of mutation at different sites on the ribosome could vary wildly and exaggerate the true distance between the superkingdoms, even if they do represent a very ancient split.

The third scenario, which we champion here, is that the bacterial ribosome evolved into an archaeal one. Again this would be a quantum evolutionary event and sequences of both rRNA and ribosomal proteins would evolve rapidly. The point we are trying to make is that these three scenarios would result in exactly the same sequence tree. Hence we must look towards independent lines of reasoning to determine which of these scenarios best describes the tree branching.

We can exclude the first scenario by comparing the structure of the ribosome in archaea and bacteria. In the 50S subunit there are six ribosomal proteins that are in the same position on the rRNA, but have non-homologous structures in archaea and bacteria [26,27]. These must have changed in at least one lineage since LUCA, regardless of LUCA's nature. Therefore, we should expect that the distance between archaea and bacteria would be exaggerated due to compensatory mutations in the rRNA and ribosomal proteins.

It is certainly reasonable to object to the third scenario because it seems implausible that a ribosome would change so much between superkingdoms, yet stay so well conserved within a superkingdom. However, there are two examples where we know that ribosome structure has indeed changed significantly. Mitochondrial ribosomes have changed dramatically from their bacterial ancestors. They have lost about half their rRNA and replaced it with additional proteins [28]. The eukaryotic ribosome evolved from an archaeal one (or technically from some sort of proto-archaeal ribsosome if the archaea are holophyletic). There are eleven ribosomal proteins found only in the eukaryotes, nine of which are conserved across the superkingdom [2], and there is good separation on rRNA trees between eukaryotes and archaea. In the two cases where the ribosome structure has changed we know it changed from another fully functional ribosome. Thus, why would it be out of the question for it to happen between archaea and bacteria? There are five ribosomal proteins present across the crenarchaea, but absent in the euryarchaea [2]. These proteins were either lost or gained in one of these groups after they split. In either case there would be a transition between two complete ribosomes. In each of these cases we can clearly see that a ribosome can undergo dramatic changes in macromolecular structure when there is proper selective pressure (or relaxation of selective pressure).

The tree presented in [29] was constructed by concatenating 31 universal proteins. 23 of these are ribosomal proteins and many more are directly involved in translation. Many taxa on the tree cluster together with high bootstrap values (greater than 80%). However, there appears to be only three connections between high level taxa that are supported with that strength. The clustering of crenarchaea and euryarchaea is well supported, as is the clustering of eukaryotes and archaea. There is also a long well supported branch between the archaeal-eukaryal clade and bacteria. We doubt it is a coincidence that these splits correspond to the greatest changes in ribosomal structure on the tree. It appears the sequence tree in [29] and rRNA trees could be merely a reflection of the large changes in ribosomal structure that have occurred throughout the true tree of life. This protein set would be expected to work better as a clock within groups that have the same ribosomal proteins. Even if ones uses more sophisticated tree building techniques, such as those in [30], the major changes in the ribosome are still going to be problematic. The authors concatenated many translational proteins and the resulting tree supported the paraphyly of archaea. Eukaryotes were placed near the archaeal species with the most similar ribosomal structures. However, a single gene tree of RNA polymerase alpha subunit (RPOA) supported holophyly in the same study. This implies some of their results are an artifact caused by structural changes in a ribosomal revolution.

The third scenario could certainly be weakened if it was found that all the ribosomal proteins were essential in bacteria and there was absolutely no way they could be tinkered with. We examined which ribosomal proteins are essential in eleven different bacterial species using the Database of Essential Genes [31]. There are sixteen ribosomal proteins that would need to be lost in the transition from a bacterium to an archaeon, as they are found across bacteria but never in archaea. None of these ribosomal proteins were found to be essential in all species, which is the first sign it is possible to lose and replace them. Four of the sixteen proteins are essential in all species except *Mycobacterium tuberculosis *(Table 1). Only four of these proteins are essential in *M. tuberculosis*, the least of any species in this data set.

**Table 1:** Essentiality of ribosomal proteins.

Species	Essential ribosomal proteins	P (ribosome is essential)	Essential but absent in Archaea	P-value
Pseudomonas aeruginosa UCBPP-PA14	32	0.5818	8	0.3373
Escherichia coli MG1655	45	0.8182	10	0.055
Bacillus subtilis 168	51	0.9273	14	0.3263
Mycoplasma pulmonis UAB CTIP	47	0.8545	10	0.0204
Francisella novicida U112	49	0.8909	13	0.2505
Helicobacter pylori 26695	29	0.5273	10	0.8493
Mycoplasma genitalium G37	51	0.9273	14	0.3263
Acinetobacter baylyi ADP1	48	0.8727	11	0.0435
Mycobacterium tuberculosis H37Rv	34	0.6182	4	0.0031
Staphylococcus aureus N315	51	0.9273	14	0.3263
Haemophilus influenzae Rd KW20	39	0.7091	9	0.1546

The essentiality of proteins that would need to be lost in the transition from an extant bacterial ribosome to an archaeal one varies from species to species. *M. tuberculosis *appears preadapted for the losses that would be necessary in the transition to an archaeon.

To determine whether this portion of the ribosome is significantly flexible we calculated a p-value assuming a binomial distribution. The essentiality of each subunit can be considered a success or a failure. The p-value measures the odds of seeing at most n essential subunits in a set of sixteen random ribosomal proteins. The odds of a random ribosomal protein being essential were estimated as the proportion of ribosomal proteins found to be essential in that species. This was done to eliminate experimental biases between the species sets, as some of the knockout experiments are more thorough than others. Several species had p-values under .05, but *M. tuberculosis *was by far the most significant with a p-value of .0031. This implies that *M. tuberculosis's *ribosome is under different selective pressure than most bacteria, and that it is the most preadapted ribosome in this dataset capable of evolving into an archaeal ribosome.

It is highly counterintuitive that nearly every universal protein could be nonessential. The difference between essential and persistent genes was discussed in [32]. The authors point out that essentiality differs in the wild and laboratory settings. Many of the ribosomal proteins listed as nonessential are still highly deleterious to lose. But the point is they can be lost under the right circumstances. It might be our proteasome centric view of the world, but we think the presence of the 20S proteasome in *Mycobacterium *could partially explain this observation. It has been proposed the major cost of mutations and mistranslation comes from dealing with mis-folded proteins [33]. The ribosomal proteins are among the most highly translated proteins in the cell, so there is lots of pressure to ensure they fold correctly. A highly advanced degradation system, like the 20S proteasome with a Pup targeting system [34], could greatly relax that selective pressure. If the initial tinkering is not lethal, one can easily imagine a scenario where compensatory mutations and structures could rapidly and significantly change the ribosome if there is proper selective pressure. We will describe such a scenario below.

It has been observed that many bacteria contain paralogs of ribosomal proteins where one form binds Zn and the other does not [35]. *M. tuberculosis *has duplicates of several ribosomal proteins, which could explain why some (but not all) of the ribosomal proteins are not essential in that genome. The authors note that thermophilic bacteria seem to prefer the Zn binding forms of the ribosomal proteins, and that there are seven Zn binding ribosomal proteins conserved across archaea and eukaryotes that are absent in bacteria. This is consistent with our ideas that major historical changes in the availability of Zn in the ocean were a significant constraint on protein structure evolution [36,37]. Bacteria vary their ribosomes to optimize for both high and low Zn conditions. One can imagine this strategy being taken to an extreme where the tweaks are not just simple displacements, but larger rearrangements. Increased availability of Zn, as the ocean became oxic, could be a factor that made toying with the ribosome favorable for the early archaea. This combined with the antibiotic pressures discussed below, could lead to a ribosomal revolution, just as the presence of two ribosomes leads to a revolution at the root of eukaryotes.

### There is a great divide in DNA replication machinery, but it can be bridged

The differences between archaeal and bacterial replication machinery is vast [4]. Leipe *et al. *claim this difference is so great that it is unreasonable to argue that one prokaryotic superkingdom evolved from the other. They list four key functions of DNA replication that are performed by completely non-homologous proteins in archaea and bacteria: the main polymerase's polymerization domain, the phosphatase that powers the polymerase, the gap filling polymerase, and the DNA primase. We will argue that the differences between archaea and bacteria do not imply the root of the tree of life has to be between them.

We must keep in mind there is some flexibility in the DNA replication machinery despite the division across the superkingdoms; consider two examples. First, many proteobacteria use a PolB family polymerase as a repair protein [38], which is almost certainly the result of HGT. Second, PolD appears to have been present in LACA, but was lost in the crenarchaea [39]. These two examples illustrate major changes in the replication machinery that occured in DNA based genomes possessing fully functional replication systems. We are arguing that an even larger event occurred between the prokaryotic superkingdoms. This event entailed viral transfers and novel innovations, but there are several proteins whose origins can be better described by vertical inheritance from the gram-positive bacteria which we review first.

Koonin *et al. *have demonstrated that many bacterial proteins have a region that is homologous to the small subunit of the archaeo-eukaryotic primase [40]. This domain is present in DNA ligase D from *M. tuberculosis*, which can act as a DNA-dependent RNA polymerase [41]. The rest of the protein is homologous to the ATP-dependent DNA ligase found in archaea and eukaryotes. Therefore, DNA ligase D is perfectly preadapted to replace the primase function of DnaG. The fission of the two halves of the protein would allow for the preservation of ligase activity while developing enhanced primase activity. A recent analysis of DNA ligases revealed many transfers between archaea, bacteria and viruses [42]. This history is very complicated, so it is hard to say with certainty where the archaeal enzymes originated. The large subunit of the primase may be a true innovation since it has no detectable bacterial homologs, but the small subunit of the primase and ATP-dependent DNA ligases both could have been inherited from the gram-positive ancestors of archaea.

The main helicase in bacteria is DnaB, while archaea use MCM6. Relevant to this discussion is the recent biochemical analysis of a protein in a prophage element in *Bacillus cereus *that has domains homologous to the MCM6-AAA domain as well as the small subunit of the archaeal primase [43]. The authors found that this protein was a functional helicase but had no primase activity. The narrow distribution of this prophage element implies its insertion was probably too recent to play a role in the origin of archaea. However, it demonstrates that there can be a selective advantage for a DNA based genome to take novel DNA handling machinery from a virus and use it in a different context. We will come back to this point later.

Bacteria use DnaA to define the origin of replication, while archaea use Cdc6. These proteins have a homologous AAA+ ATPase domain, but have little similarity otherwise. However, the bacterial protein RuvB has the same domain combination as Cdc6. RuvB, Cdc6, and DnaA were all put in the same superfamily in a recent classification of AAA+ domains [44]. RuvB is recruited to Holliday junctions by RuvA where it forms a hexamer around the DNA [45], just like Cdc6. It is plausible that Cdc6 evolved from RuvB.

Archaea use a protein called Hjc to resolve Holliday junctions instead of the bacterial RuvABC system. Hjc is related to the alternative bacterial system RecU [46]. The only bacteria that use RecU are the firmicutes, and they also have RuvABC. We argue below archaea are derived from within the firmicutes. It is possible that the redundancy in Holliday junction systems allowed RuvB to drift in function. The homology between RecU and Hjc could be explained by the presence of a Holliday junction resolvase in LUCA under the canonical rooting. However, if the hypothetical RNA-DNA hybrid LUCA proposed in [4] was dealing with Holliday junctions we argue it probably would also need topoisomerases at that point. However, since the distribution of topoisomerases is different across the prokaryotic superkingdoms [47,48] that would imply the ancestral topoisomerase was displaced in at least one lineage. This weakens the proposal in [4]. We feel it is more likely archaeal topoisomerases evolved from bacterial ones as Cavalier-Smith has proposed [16].

There are certainly large differences between archaeal and bacterial DNA replication machineries. We have demonstrated the divide between replication systems has some flexibility, and this opens the door for a replication revolution. It is possible to come up with detailed scenarios for how each of the archaeal replication proteins originated. These results are summarized in Table 2. We will elaborate on this **s**cenario below. However, there are several archaeal replication proteins that do not appear to have any homologs in bacteria; namely histones, PolD, and the large subunit of the archaeal-eukaryal primase. These are true innovations, but there really are not that many of them; certainly not enough to make the transitions seem unreasonable in light of the polarizations presented above.

**Table 2:** Summary of differences in DNA replication machinery of Archaea and Bacteria.

Function	Superkingdom	Protein	PDB ID (if applicable)	SCOP Superfamily Combination	Proposed Origin in Archaea
Origin recognition	Bacteria	DnaA	1l8q	_gap_,52540,48295	
	Archaea	Cdc6/ORC (single or multiple homologues)	1fnn	52540, 46785	evolved from RuvB
Replicative helicase	Bacteria	DnaC	3ec2	52540 *	
	Archaea	MCM	3f9v		viral transfer
Helicase loader	Bacteria	DnaB	1b79 (n-terminal domain)	gap, 48024	
ssDNA-binding protein	Archaea, Bacteria	SSB (one subunit)	1o7i	50249	archaeal SSB evolved from bacterial SSB
	Archaea	RPA (one or three subunits)	2b28	50249, 50249, 50249	evolved from SSB
Primase	Bacteria	DnaG	1dd9	56731	
	Archaea	PriA (small)	1zt2:a	56747, gap	viral transfer or vertically inherited
	Archaea	PriB(large)	1zt2:b	140914	innovation
Replicative Polymerase	Archaea, *Bacteria*	PolB (one or multiple homologues)	1q8i	gap, 56672	viral transfer
	Archaea	PolD (small)	none	gap, 56300	innovation
	Archaea	PolD (large)	none	gap	innovation
	Bacteria	PolC (DnaE)	2hqa	89550, large gap*	
DNA sliding clamp	Archaea	PCNA (three subunits)	3a2f	55979, 55979	evolved from β-clamp
	Bacteria	β-clamp (dimer)	1ok7	55979, 55979, 55979	
Clamp loader	Bacteria	γ-Complex (three subunits)	1jr3:a	52540, 48019	
	Archaea	RFC (two subunits)	1iqp	52540, 48019	evolved from γ-Complex
Removal of primers	Archaea	Fen1	1rxw	88723, 47807	viral origin
	Bacteria	RNase H	1jl1	53098	
	Archaea	RNase HII	1eke	53098	archaeal RNAase HII evolved from bacterial RNAase HI
	Bacteria	PolA	2kfz (mssing c-terminal domain)	53098, 56672	
	Archaea, *Bacteria*	DNA ligase(ATP-DEP)	1xn9 (human)	117018, 56091, 50249	viral origin or vertical inheritance
	Bacteria	DNA ligase(NAD-DEP)	1dgs	56091, 50249, 47781	
Preinitiation complex	Archaea	gins	2eho(human)	158573	true innovation

The list of protein functions was compiled from box 1 in [20] and table one in [129]. Italics indicate a probable horizontal transfer to a superkingdom. There are very few proteins in archaea that are true innovations. Many of their unique replication proteins could be recruited from bacterial or viral systems. A * indicates the Superfamily database was used to predict domain assignments of PDB entries not yet classified in SCOP.

The proposal of two independent inventions of DNA replication has recently been challenged [49]. The authors argue that ribonucleotide reduction is thermodynamically unfavorable, so convergent evolution is highly unlikely. They note that all ribonucleotide reductases have been shown to have a monophyletic origin. Finally, they argue that the proteins that are universally conserved imply a high fidelity replication system in LUCA that could not have been RNA based. The hypothesis that the root must be between the superkingdoms is diminished when one combines these arguments with the scenarios we have outlined here.

### Are the Archaea Paraphyletic or Holophyletic? We're agnostic

So far we have presented several independent arguments that strongly polarize archaea as a taxa derived from within bacteria. We have demonstrated that although there are vast differences between the ribosomes and DNA replication machinery between the prokaryotic superkingdoms, none of the arguments associated with their respective proteins seems insurmountable. We will soon present a novel hypothesis to account for this; but first we must pinpoint the bacterial roots of archaea. One cannot properly reason about this rooting without first discussing whether archaea are paraphyletic (eukaryotes branch within them) or holophyletic (eukaryotes are their sisters). As there is clearly a relationship between archaea and eukaryotes it is vital to differentiate between these two scenarios to understand their origins. We will review the current available data, and argue that for now precise agnosticism seems the best course, that is, any hypothesis on the origin of archaea must accommodate both models. That said, we lean towards holophyly and our hypothesis does as well.

Eukaryotes and Archaea are sister clades under the standard three domain model. However, James Lake proposed that eukaryotes had a crenarchaeal (eocyte) origin based on a shared indel in EF-1 and similarities in their ribosomal structures [13,14]. This hypothesis never gained much support because there was little phylogenetic evidence to corroborate it. However, recent work [30] has shown that there is sequence data that implies archaea are paraphyletic and eukaryotes have a crenarchaeal-like ancestor. Conversely, another analysis done around the same time supported a deep branching archaeon as the host of mitochondria [50], which would be inconsistent with the eocyte hypothesis. They demonstrate that eukaryotes inherited both crenarchaeal and euryarchaeal specific proteins, so ancestry from either group alone is not enough to explain the eukaryotic protein repertoire. However, several deep branching archaeal genomes from korarchaeota and thaumarchaea are now available and change the context of some of these conclusions [51,52]. Both of these groups appear to contain a mix of crenarchaeal and euryarchaeal genes so the observation in [50] could be explained by a member of one of these groups being ancestral to eukaryotes.

Cavalier-Smith's hypothesis on the origin of the neomura relies on a sisterhood relationship between archaea and eukaryotes [16]. As discussed below, he mainly roots the neomura using traits actinobacteria share with eukaryotes, but not archaea. This only makes sense if archaea and eukaryotes are sisters, otherwise the traits should be present in at least some archaea. He lists eight properties that are unique and ubiquitous in archaea [16]. All of these traits strongly imply that archaea are monophyletic. However, most of them do not differentiate between whether archaea are holophyletic or paraphyletic.

For instance, the unique isoprenoid ether lipids found in all archaeal membranes are best explained by their presence in LACA. Eukaryotes have lipids that are more similar to those of bacteria. It would be more parsimonious for archaea and eukaryotes to be sisters with a single change in lipid structure. Any other scenario requires a reversion in eukaryotes back to the bacterial state. Even though this is not parsimonious, it is not out of the question because the mitochondrial ancestor would have all the necessary genes to make bacterial membranes [53]. We have to admit that does not seem unreasonable relative to the innovations we are discussing in this work. This certainly seems like a case where simple parsimony in terms of any one trait, even membrane structure, will be misleading.

The only one of these properties that appeared really informative in regard to this problem was the split gene for RPOA. RPOA is the only single gene tree that supported the three domain model in [30], so it is clear eukaryotes did not get this protein from the mitochondrial ancestor. Reassembling the split gene is highly improbable, so there is no reason to doubt the fused genes are monophyletic. This strongly contradicts the original eocyte hypothesis. However, novel genomic data has revealed that representatives from the deep branching phyla korarchaeota and thaumarchaeota have the non-split form of this gene [51,52]. This opens the door for a more specific version of the eocyte hypothesis where eukaryotes stem from either of these groups. Therefore, we have examined what additional data have to say about these taxa. The branch order between them has not yet been resolved, but it appears safe to assume they both branch before the split between crenarchaea and euryarchaea. This branching is supported by several phylogenetic trees as well as the non-split RPOA. This assumption will be key to our subsequent reasoning in several ways.

It seems impossible to come up with a scenario utilizing all the traits we discuss below that is completely parsimonious for all traits at the same time. With that in mind we have tried to reason which traits can be better explained by convergent evolution than others. When we observe convergent evolution happening at an indel site we do not consider it informative. Independent loss in any form is much easier than independent invention. Loss seems to be the rule rather than the exception in archaea. Both the thaumarchaeota and korachaeaota have traits that were thought to be specific to either euryarchaea or crenarchaea. For instance euryarchaea use FtsZ for cell division while crenarchaea use the cdvABC system. Intriguingly the thaumarchaeotal genomes have orthologs of both of these systems [54]. This implies that the crenarchaea and euryarchaea each lost one of these systems. This is not the most parsimonious solution, but it is the only one that is consistent with the apparent branch order of these taxa. Many other traits have the same distribution pattern. It is clear that groups of archaea can lose proteins of major functional importance. We will attempt to address these distributions in our hypothesis below.

Beyond the EF-1 indel that implies paraphyly, six highly conserved indels were found to be informative in describing the relationship between archaea and eukaryotes in [50]. The authors only looked at derived insertions with well conserved sequences. The authors state that four indels argue for the holophyly of archaea. There is one indel that is shared between eukaryotes and crenarchaea, as well as one shared between the euryarchaea and eukaryotes. This implies there was a reversion in at least one lineage or a horizontal transfer.

We have analyzed those six indels as well as EF-1 in the context of the recently sequenced deep branching genomes (Table 3). Only the indels that differ between archaeal groups are useful for determining their branch order. Therefore we only created alignments that contained archaeal sequences to ensure these indels were not artifacts created by including eukaryotic and bacterial sequences. Where possible we also used structural alignments from representatives of the superkingdoms to further ensure the larger indels were real (a similar methodology as used in [11]).

**Table 3:** Analysis of potentially informative gene structures in korarchaeota and thaumarchaeota.

Sequence Property	Initially reported to support	In Korarchaeota	In Thaumarchaea	Now Supports	Notes
Split RpoA	Holophyly	X	X	Holophyly or paraphyly (Thaumarchaeota or Korarchaeota)	

7 AA Insert in EF-1	Paraphyly (Crenarchaea)	2 aa insert shared with thermoplasma	X	Paraphyly, but weakly	Must be reversion in Euryarchaea

6 AA insert in RadA	Paraphyly (Euryarchaea)	?	?	?	There is probably an artifact in the original alignment.

1 AA insert in SecY	Paraphyly (Crenarchaea)	absent	X	Paraphyly (Crenarchaea or Thaumarchaeota)	Single Glycine, but anchors are really nicely conserved

1 AA insert in proAS	Holophyly	X	absent	Probably holophyly, maybe paraphyly (Thaumarchaeota)	BLAST reveals Thaumarchaeota may have HGT from Firmicutes.

7 AA insert in GatD	Holophyly	X	X	Inconclusive	Completely conserved, but its not clear the Eukaryotes inherited this from Archaea

2 AA insert in PBG	Holophyly	Gene is absent	Gene is absent	Inconclusive (bacterial origin)	This protein is present in bacteria, so the Thaumarchaeota and Korarchaeota probably lost it

2 AA insert in ribosomal S12	Holophyly	X	X	Inconclusive	It is conserved across the Eukaryotes and Archaea

Each indel was analyzed by creating an alignment of archaeal sequences from BLAST searches. We consider these results to be inconclusive until thaumarchaeota and korarchaeota are sampled better.

First, the reported indel shared between euryarchaea and eukaryotes in the DNA repair protein RadA appears to be an artifact. The euryarchaeal and crenarchaeal sequences align well in the indel region (Additional File 1; Figure S1). This is important because it was the only line of evidence in that work that implied a relationship between euryarchaea and eukaryotes. This new alignment, in conjunction with the split RPOA gene, implies eukaryotes either descend from within the deep branching archaea or are their sisters.

We also argue that the two reported indels in the alignments of Beta-glucosidase/6-phospho-beta-glucosidase/beta-galactosidase (PBG) and ribosomal protein S12 are both uninformative based off the authors' own analyses (supplemental data from [50]). The indel in ribosomal S12 is conserved across all archaea and eukaryotes, so it implies nothing about their branch order. The indel in PBG is uninformative because the authors conclude the eukaryotic version of this gene is probably of bacterial origin (supplemental data from [50]). Therefore, the state of the gene in archaea implies nothing about the branch order of these groups.

Two of the remaining four indels are only a single residue. The glycine insertion in SecY is present in thaumarchaeota and eukaryotes, but absent in korarchaeota. That weakly implies a relationship between eukaryotes and thaumarchaeota. However, given that the insertion is present in some of the deep branching taxa, but not in all euryarchaea, implies there was at least one secondary loss of this insertion. This is reasonable since the insertion is a single glycine residue, and will not have a dramatic effect on protein structure.

The single residue insertion in prolyl-tRNA aminoacyl synthetase initially implied archaea were holophyletic, however, the insert is missing in the thaumarchaeal genomes. When these genes are used to seed a BLAST [55] search they hit firmicutes more so than other archaea. This implies a possible horizontal transfer to thaumarchaeota. If so this insert could still support holophyly, but that cannot be concluded with absolute certainty.

This leaves us with two larger indels in EF-1 and glutamyl-tRNA amidotransferase subunit D (gatD). The seven AA insert in gatD is well conserved in the archaeal alignment. A structural alignment with a bacterial homolog reveals this indel is not an artifact caused by the sequence alignment (data not shown). The phylogenetic tree for this family (presented in the supplemental data of [50]) places archaea and eukaryotes as sisters with 100% bootstrap support. This is remarkable because the archaeal proteins have a different domain combination and quaternary structure than the eukaryotes and bacteria [56]. However, it seems that tree is too good to be true. We have attempted to verify the history of this indel, and found that the tree in [50] was missing a bacterial paralog. *E. coli *has members of two paralogous families of l-asparaginases [57], and it appears only one of them was present in the initial tree. The tree in Additional File 2; Figure S2 shows that fungi and the rest of the eukaryotes received the same domain superfamily from two distinct sources. Their sequences are mixed in with some bacteria, which implies there were some recent horizontal transfers. This tree is not well resolved, but it certainly does not support the notion eukaryotes inherited this protein from their archaeal ancestor. That, as well as the differences in domain combination and quaternary structure, implies this indel is inconclusive with regards to holophyly verses paraphyly.

**Figure 2:**
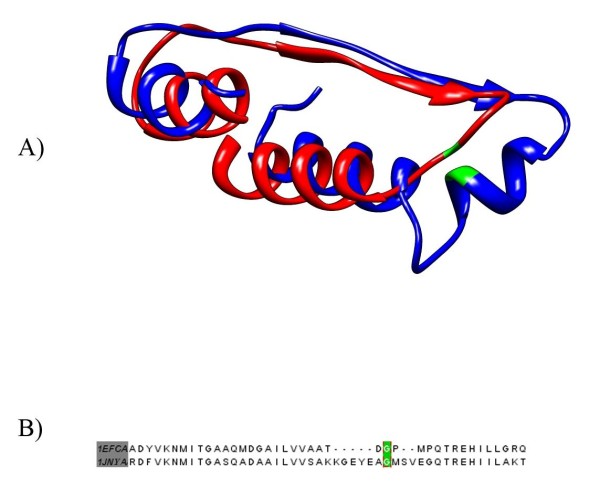
**Structural alignment of EF-1 and EF-Tu**. The structural alignment of EF-1 (1JNYA) and EF-Tu (1EFC) in A, and the corresponding sequence alignment in B, show the potential for two independent indels in this region that confounds analysis.

EF-1 also appears inconclusive. The insert shared between crenarchaea and eukaryotes is present in thaumarchaeota, but not korarchaeota. Our alignment revealed there are actually four different forms of indel at this site in archaea (Additional File 3; Figure S3). This implies there is some plasticity in this region in archaea. This is in contrast to the bacterial alignment which has no indels in this region. A structural alignment between a bacterial representative from *E. coli *and an archaeal one from *Sulfolobus solfataricus *reveals the conserved glycines in the sequence alignments are very close in their position in both forms of this indel (Figure 2). It is possible there were two insertions near the root of archaea that preserved the position of that residue. This indel's history does not appear to be parsimonious, which weakens it usefulness as a marker. Therefore, this indel appears to weakly support archaeal paraphyly, but we consider it inconclusive.

The ribosomal proteins are the other side to this story. In a previous study, five ribosomal proteins were found in at least one crenarchaeon, but not in any of the euryarchaea (L38e, L13e, S25e, S26e and S30e) [2]. These, as well as four others that are not universal in archaea, are conserved across eukaryotes. We examined what ribosomal proteins are present in the thaumarchaeal and korarchaeal genomes (Table 4). It still appears that Lake is correct that crenarchaea have more similar ribosomal proteins to eukaryotes than any other group of archaea.

**Table 4:** Informative ribosomal proteins in thaumarchaeota and korarchaeota.

Ribosomal protein	Pfam	Eukaryotes	Bacteria	Crenarchaea	Euryarchaea	Korarchaeota	Thaumarchaeota
L38a	PF01781	182	0	7	0	0	0

L13e	PF01294	202	0	19	0	1	0

S25e	PF03297	195	0	20	0	1	0

S26e	PF01283	189	0	23	1	1	3

S30e	PF04758	179	0	22	0	1	3

L20a		No hits	No hits	Hits	Hits	No hits	No hits

L35ae	PF01247	172	0	5	10	0	0

L14e	PF01929	196	0	16	8	1	0

L34e	PF01199	208	0	21	21	1	0

L30e		No hits	No hits	hits	hits	Hits	No hits

This table was constructed from [2]. The values listed were taken from searches of the Pfam website. Ribosomal proteins L20A and L30E were not well defined in Pfam so BLAST searches were performed instead. These results support the eocyte hypothesis, but it is plausible that there were independent losses of ribosomal subunits in archaea based on additional data.

The korarchaeota are missing three ribosomal proteins found in some crenarchaea and eukaryotes. They have five ribosomal proteins that are present across eukaryotes that are absent in thaumarchaeota. There are two ways we can interpret this trend. If archaea are paraphyletic then this distribution is best explained by the invention of ribosomal proteins after LACA. LECA could branch between the korarchaeota and crenarchaea, before the RPOA gene split. The alternative interpretation is that archaea are holophyletic and the archaeal ancestor had all the ribosomal proteins that are in any archaeon and at least one eukaryote. There would have to be several independent losses of each these ribosomal proteins. Again this is not parsimonious, but there is evidence it has occurred several times so we must consider it. Again, it can be argued that if a protein is present in korarchaeota and crenarchaea, but absent in euryarchaea, it must have been lost. The archaeal ribosomal proteins are more dispensable than their counterparts in the other superkingdoms [2], so they might not be a reliable marker for rooting eukaryotes in archaea.

For now it seems the only reasonable stance in light of all of this evidence is agnosticism. Only when thaumarchaeota and korarchaeota are sampled better, and their positions in the archaeal tree are determined robustly, will it be possible to state with confidence whether archaea are holophyletic or paraphyletic. We might always be left trying to weigh whether reversion of ribosomal proteins or indels is the more parsimonious scenario. However, several of these traits clearly exclude the root of eukaryotes from within crenarchaea and euryarchaea. Therefore, any hypotheses on the origin of eukaryotes that invokes specific taxa within those groups can be rejected with confidence (for a discussion of the many hypotheses on this subject see [58]). However, it may be possible those scenarios could be reworked to fit thaumarchaeota or korarchaeota once they are sampled better.

### Weakening the neomuran hypothesis

Now that we have argued for the true distance between the superkingdoms we can begin to address how it could be bridged. From our discussion above we feel we must be cautious about declaring the debate closed on the holophyly of archaea. Therefore, we are more interested in traits shared between a group of bacteria and all archaea than those shared with eukaryotes. Cavalier-Smith has presented fourteen reasons why the root of the neomura is probably within or next to actinobacteria [16]. Two of these traits are shared between actinobacteria and neomura, but the other twelve are only shared between eukaryotes and actinobacteria. Under this scenario these twelve traits would be lost in the ancestor of archaea, which implies archaea are holophyletic. We will review these fourteen traits, and argue that placing the archaeal ancestor in the bacilli makes more sense. We use the term neomura to refer to the clade of eukaryotes and archaea, but when we refer to the neomuran hypothesis we refer to Cavalier-Smith's rooting of that clade in the actinobacteria.

The first piece of evidence that places the neomuran root near actinobacteria is the proteasome. Actinobacterial and archaeal 20s proteasomes are well separated on phylogenetic trees which implies the presence of the 20s proteasome across these groups is not the result of recent horizontal transfers. Recently 20S proteasomes have also been found in sequenced genomes from verrucomicrobia [59] and leptospirillum metagenomic sequences [60]. This somewhat weakens the actinobacterial argument for ancestry, as archaea could have inherited a proteasome from these other groups. However, these recent findings do not weaken the polarization argument; it just excludes the root from these additional groups.

The second trait apparently shared between actinobacteria and all neomura is the post translational addition of CCA to the 3' end of tRNAs. The gene performing that function in archaea is tRNA CCA-pyrophosphorylase (protein cluster PRK13300 [61]). One of the domains, PAP/Archaeal CCA-adding enzyme, does not hit any bacteria in the Superfamily database [62]. Since the CCA addition is performed by nonhomologous enzymes this is not strong evidence for rooting neomura. There is also an analogous enzyme conserved across bacilli (protein cluster PRK13299). Even if archaea inherited this function from their bacterial ancestors, it is not clear which gram-positive group provided it.

Now we must address the remaining dozen traits shared between actinobacteria and eukaryotes. Although there were initial reports of sterol synthesis in the actinobacteria [63,64], the latest work has found no evidence for a complete pathway [65]. The authors report that the few cases of the full pathway in bacteria (all outside the actinobacteria) are probably the result of horizontal transfer. However, they find several sterol synthesis enzymes are present in many actinobacteria. They conclude these are probably the result of a transfer from eukaryotes, but this is not supported by their trees, which show good separation between eukaryotes and actinobacteria. Several sterol enzymes appear to have been inherited vertically from actinobacteria to eukaryotes. This is certainly consistent with Cavalier-Smith's hypothesis. This is a good example of the dangers of closing the debate on the position of the root too soon. Their trees clearly support an alternative hypothesis, but that data is buried in the supplemental material without discussion of the opposing view.

Initial reports also claimed the presence of chitin in actinobacteria [66]. However, there is no gene for chitin synthase in actinobacterial genomes. Several of them have chitinase which breaks chitin down. Also, chitin is found in metazoa and fungi, but not in archaeplastida which implies this enzyme was not in LECA.

It is true that actinobacteria have many serine/threonine signaling systems related to cyclin-dependent kinases [67]. This would be a key preadaptation to the cell cycle. However, it has recently been shown that *Bacillus subtilis *also has an extensive network of such regulation [68]. Therefore this line of evidence is consistent with either gram-positive group being ancestral to neomura.

Phosphatidylinositol is an interesting case. Recent work on this subject confirms the presence of phosphatidylinositol synthase as well as the eukaryotic form of cardiolipin synthase in many actinobacteria [69]. These enzymes are paralogs. We could not create a quality tree for this superfamily because the alignment was of low quality. However BLAST searches showed a good separation between prokaryotic and eukaryotic sequences that implies this is not the result of a recent HGT. It is difficult to determine exactly what family each prokaryotic homolog belongs to, so it is hard to say with certainty what other groups of bacteria have phosphatidylinositol. It is certainly possible eukaryotes inherited phosphatidylinositol from actinobacteria.

Some actinobacteria do have an α-amylase with similar primary structure to the form found in metazoa, but a recent comprehensive study found several other bacteria that did as well [70]. The authors concluded this was probably the result of a horizontal transfer due to their position in the phylogenetic tree as well the extremely sparse distribution of this form in actinobacteria. Therefore, this is not evidence for actinobacterial ancestry of the neomura.

**Figure 3:**
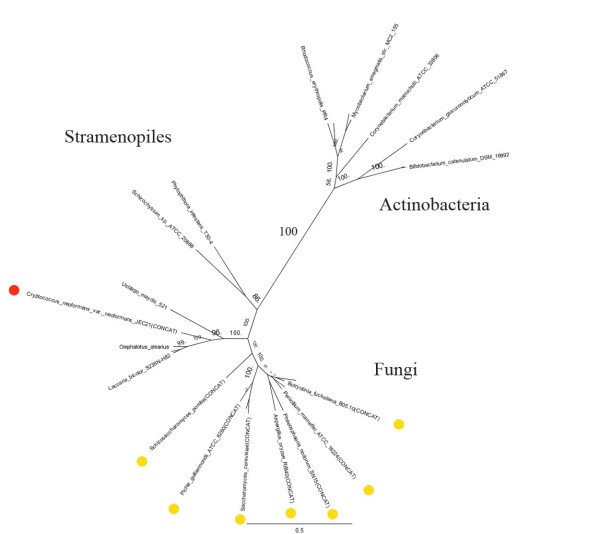
**Maximum likelihood tree of fungal type Fatty Acid Synthase (FAS) complex**. This tree implies eukaryotes did not get FAS from a recent transfer, but it is also not clear whether or not it was in LECA. Circles indicate the split form of the gene. This gene is split in two different places in the fungi indicated by the yellow and red circles.

The fatty acid synthetase (FAS) complex found in actinobacteria is unique among bacteria in that it is the same form as found in some fungi [71]. These fungi have the FAS complex split into two genes, but actinobacteria have it fused. Our phylogenetic trees are consistent with actinobacterial ancestry (Figure 3). However, the distribution of the fungal type complex in eukaryotes does not conclusively prove that this enzyme had to be in LECA. The only group outside the Fungi with this complex are stramenopiles. However, the animal type FAS is also present in some alveolata, so there could be some functional displacements. Actinobacteria probably played a role in the evolution of this enzyme in eukaryotes, but not necessarily via the neomuran hypothesis.

The argument that the exospore structure of actinobacteria could be a precursor to eukaryotic spore structures seems sound [72], but we are unable to locate a list of proteins involved in exospore formation. Without specific proteins homologs we cannot begin to evaluate this with bioinformatics. However, this argument becomes irrelevant if one invokes a viral ancestor of the nucleus as in [73].

Cavalier-Smith has also suggested that the C-terminal HEH domain found in the Ku proteins of some actinobacteria is ancestral to the HEH domain found in the eukaryotic Ku70 protein. However, the sequence analysis in [74] conclusively demonstrates eukaryotes did not inherit the HEH domain from actinobacteria. This domain is very compact and common. Therefore, it is not out of the question that it was recruited twice to the C-terminus of similar structures. Consequently we do not take this as evidence that eukaryotes inherited Ku from actinobacteria.

Several traits initially listed as unique to actinobacteria are now found in enough other bacterial groups to now be considered ambiguous markers. Actinobacteria do have tyrosine kinases, but they have recently been put into a bacterial specific family, BY-kinase [75]. This family is present across actinobacteria, firmicutes, and proteobacteria, so it is does not support an actinobacterial rooting exclusively in the neomura. Many groups of bacteria have HU (histone H1 homologs) according the Superfamily database. This protein is relatively short, so we should not expect sequence to resolve its history. It is possible this protein was inherited from actinobacteria, but there are too many other possibilities to state that with certainty. Calmodulin-like proteins are now found in many bacteria, so this trait is not specific enough to root neomura near actinobacteria as Cavalier-Smith now admits [8]. The Superfamily database reveals that trypsin-like serine proteases are present in many groups of bacteria, but absent in archaea. This appears to be another trait that is too general to be useful for rooting neomura.

### Evidence that supports a firmicute ancestry for archaea

Skophammer *et al. *compiled several reasons to argue archaea are derived from bacilli [12]. There is an insert in ribosomal protein S12 that is present in archaea and bacilli (and maybe chloroflexi). Skophammer *et al. *conclude this indel is derived, but we argue elsewhere this polarization is flawed [11]. The insertion appears well conserved between archaea and bacilli regardless of whether it is ancestral or derived.

Skophammer *et al. *also note that there is a shared deletion between firmicutes and archaea in PyrD. Our own work strengthens this connection by considering the quaternary structure of PyrD. The form that has the deletion also has an additional subunit, PyrK. The sequence and structure of the firmicute PyrD 1B are both shared by archaea. Our phylogenetic analysis of this protein implies this is not the result of recent horizontal transfers [11].

Skophammer *et al. *note that many enzymes involved in the biosynthesis of unique archaeal membranes have previously been found in firmicutes [21]. The isoprenoid lipid precursors of archaeal membranes are made via the mevalonate pathway, which is five enzymes long. The KEGG database [76] reveals the entire mevalonate pathway is present in several bacilli as well as some actinobacteria (KEGG module M00191). The unique stereochemistry of archaeal membranes is determined by the enzyme geranylgeranylglyceryl phosphatase. Homologs of this enzyme are present in bacilli (protein cluster PRK04169), but appear to be absent in actinobacteria. The authors of an analysis of archaeal membrane biosynthesis propose that archaea became genetically isolated from bacteria once their membrane chemistry changed [77]. They suggest that archaea branched early from within bacteria, but their hypothesis is also consistent with a later gram-positive origin. Cavalier-Smith's own analysis [8] suggests that eukaryotic enzymes that make n-linked glycoproteins, which are necessary for the loss of peptidoglycan, evolved from the firmicute specific gene EspE. Therefore, for several reasons, the firmicutes are the bacterial group most preadapted to gain archaeal membranes.

**Figure 4:**
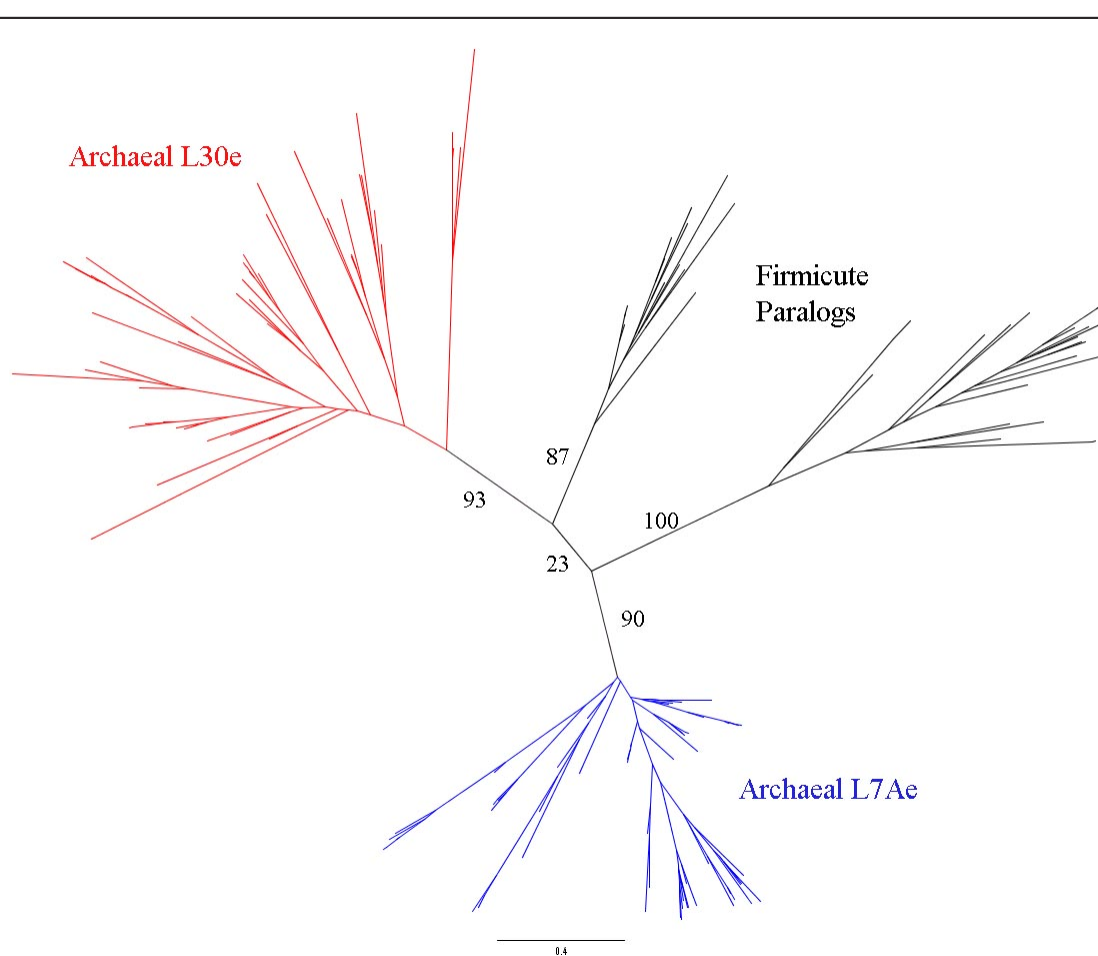
**Alignment of L7Ae paralogs in archaea and firmicutes**. This tree is consistent with a firmicute origin for two archaeal ribosomal proteins.

Homologs to ribosomal proteins L30e and L7ae are found across firmicutes. This is evidence of the link between firmicutes and archaea. Pfam [78] shows this family in several other groups, but many firmicutes contain two copies of this family. One of these paralogs has been characterized as a ribosomal protein, but neither is essential [79]. We constructed phylogenetic trees to see if they are consistent with vertical inheritance (Figure 4). There is good separation between the paralogs in firmicutes, which implies the duplication occurred early in firmicutes. All archaeal and eukaryotic genomes contain at least two copies of this family. The phylogenetic tree of the archaeal and firmicute sequences places the firmicute paralogs between the archaeal paralogs. The firmicute sequences are paraphyletic, albeit with very weak support. If these proteins are the result of independent duplications the archaeal sequences should cluster together, not appear on opposite ends of the tree. However, it is possible one of the archaeal sequences evolved rapidly after duplication.

One of the paralogs in *Bacillus subtilis *was found to localize to a different portion of the ribosome than either of the archaeal paralogs [79]. The proteins would not only have to jump superkingdoms for a transfer to occur, they would also have to bind to a different region of the rRNA without interfering with ribosome assembly. We argue it would be less disruptive for a protein already present to gradually bind a different piece of rRNA. The separation between the superkingdoms in the phylogenetic trees also argues against HGT. If this is the result of vertical inheritance only two possibilities explain it. Either the firmicutes are ancestral to archaea, or the root lies between archaea and firmicutes. Our polarization of PyrD 1B's quaternary structure eliminates the latter rooting as a possibility. Thus this tree appears to support a firmicute ancestry for archaea, although it may just be the result of rapid evolution of structures in different contexts in the ribosome.

As discussed above, almost all the firmicute genomes have a unique Holliday junction resolvase, RecU, which is only found sparsely in other bacterial groups. It is homologous to the archaeal Holliday junction resolvase, Hjc [46]. Therefore the firmicutes have a DNA repair mechanism more similar to archaea than any bacterial group.

Hsp90 is missing in all archaeal genomes, so its presence across eukaryotes and bacteria implies it was inherited from the mitochondrial ancestor. However, a detailed analysis of this family did not reveal a relationship between eukaryotic and proteobacterial sequences [80]. Instead, the eukaryotic sequences branch within the gram-positive bacteria. The authors argue this supports the classical neomuran hypothesis, but eukaryotes are sisters to firmicutes rather than actinobacteria in that tree (albeit with moderate support). This would slightly favor firmicutes over actinobacteria ancestry. In either case it supports the view that the archaeal ancestor lost Hsp90.

### Peroxisomes: the red herring?

There are several traits present in either firmicutes or actinobacteria that argue they are ancestral to either eukaryotes or archaea. The only trait that argues actinobacteria are ancestral to the neomura is the proteasome. Several more traits make compelling arguments that actinobacteria are ancestral to eukaryotes, but certainly not the dozen traits listed in [16]. In Cavalier-Smith's most recent version of the neomuran hypothesis he concludes firmicutes contributed a significant number of genes to the neomuran ancestor [81]. He proposed neomura originated as sisters of actinobacteria, and both of these taxa are descendents of firmicutes. That proposal is dependent on his argument that actinobacteria are derived from firmicutes, which is one of the less developed ideas in [8]. We believe he is wrong in his assertion that our analysis of the indel in ribosomal S12 [11] does not support firmicute ancestry of archaea. It is only shared (and well conserved) between bacilli and archaea regardless of the polarization of that indel. Cavalier-Smith is also not aware of the arguments about L7AE paralogs and RecU we present here for the first time. So we are left with a stronger list of reasons supporting firmicute ancestry and a weaker list for actinobacterial ancestry. However, there are still some key eukaryotic proteins that appear to have descended from actinobacteria. We will try to reconcile this apparent anomaly.

The peroxisome is an organelle with a single membrane, found across eukaryotes, that has various oxidative functions including the synthesis of some lipids [82]. They have been observed to divide independently of the rest of the cell, which initially led someto question whether they had an endosymbiotic origin [83,84]. Two recent studies both concluded that the peroxisome was likely derived from the endoplasmic reticulum [85,86], which led those initial proponents of peroxisomal endosymbiosis to abandon that idea.

However, [85] found that many peroxisomal proteins likely originated in cyanobacteria, α-proteobacteria, or actinobacteria. The authors suggest that the proteobacterial genes were probably transferred from the mitochondria, which is consistent with observations that mitochondrial genes are often retargeted to other organelles [87]. However, recent work argues for an endosymbiotic origin of the peroxisome from an actinobacterium [88]. These latter authors demonstrate that at least two proteins imported into the peroxisome are of actinobacterial origin, and that the peroxisomal proteome has higher average BLAST scores to actinobacteria than any other group of prokaryotes. They argue that the retargeting of mitochondrial proteins after their genes migrate to the host's genome is easier than *de novo *targeting of peroxisomal proteins. They propose this masks the true history of the peroxisome.

The literature proposes two scenarios to explain the origin of the peroxisome: either the peroxisome was an endosymbiont, or actinobacteria were not endosymbionts. Clearly there is a third possibility; there was an actinobacterial endosymbiont, but the peroxisome is not a descendent of that membrane. That is to say, genes of an endosymbiotic origin were targeted into the peroxisome, but historically they are foreigners there. How could this be? A primitive peroxisome derived from the endomembrane system would be beneficial because it would separate dangerous oxidative chemistry from the rest of the cell. Proteins would be targeted to the organelle with relative ease since that system would be developed through mitochondrial endosymbiosis. Genes would be copied from the actinobacterial endosymbiont to the host genome (but not necessarily lost in the actinobacterium), and then imported into the peroxisome. This would be advantageous because some of these reactions would do better in that specialized environment rather than their original host. Potentially there would be less cost involved in maintaining an organelle that already existed versus an entire endosymbiont. Once enough genes were present in the host, the actinobacterial endosymbiont would essentially be a parasite, and complete gene loss would be beneficial.

Contrast the peroxisome to organelles such as plastids and mitochondria which retained both genomes and membranes long after they became organelles. Some have questioned why some organelles retain any genes at all [89]. These authors note that most genes retained in plastids and mitochondria are membrane-spanning proteins involved in core photosynthetic and respiratory systems. They agree with an earlier proposal that these proteins must be kept in the organelle to be able to quickly respond to, and balance, redox gradients [90]. In other words, plastids and mitochondria have retained membranes and genes because their functions are centered on membrane based chemistry. The stripped down endomsymbionts perform these functions better than a novel organelle initially could, so they are left with a few essential genes and membranes they inherited from endosymbiosis. These genes come with a high cost because the organelles need to import the machinery to translate them as well as the machinery to replicate the genes that encode them. Therefore one can hypothesize that other endosymbionts whose functions are not as membrane-centric could be replaced by organelles that are not of endosymbiotic origin. Unfortunately, plastids and mitochondria have shaped our expectations that endosymbionts will leave both membranes and genomes behind. We believe this is an over simplistic expectation.

We argue actinobacterial endosymbiosis accounts for the traits shared between eukaryotes and actinobacteria, as well the phylogenetic trees that place actinobacteria as sisters of the peroxisomal proteins. The fact that numerous mitochondrial proteins are imported into the peroxisome is evidence this endosymbiosis occurred after mitochondrial endosymbiosis. This would reconcile the apparently conflicting signals in terms of which gram-positive group is ancestral to archaea and eukaryotes. We find this scenario more reasonable than invoking an extinct lineage of gram-positives that has all the traits listed in Table 5 and Table 6. However, if a genome is sequenced that contains actinobacterial specific traits as well as firmicute specific traits listed here we would have no need to invoke endosymbiosis. It is also possible to reconcile the canonical rooting with the traits shared by actinobacteria by invoking this endosymbiotic hypothesis.

**Table 5:** Summary of data used to support actinobacterial ancestry of archaea.

Trait	Supports actinobacterial ancestry of Neomura	Supports actinobacterial Ancestry of Eukaryotes	Exclusive to Actinobacteria among Bacteria
20s proteasome	Yes	Yes	In a few other bacterial genomes

CCA added post-transcriptionally	No	No	Also in Firmicutes

Sterols	No	Yes	In a few other bacterial genomes (HGT), but only ones that could be vertical are actinobacterial

Chitin	No	No	Not in Actinobacteria

Serine/threonine signaling system	No	Yes	In Firmicutes too

Tyrosine Kinase	No	Yes	In many bacteria

H1 linker histone	No	Yes	In many bacteria

Calmodulin	No	Yes	In many bacteria

Phosphatidylinositol	No	Yes	Apparently unique to Actinobacteria, but related enzymes in other groups

Serine proteases	No	Yes	In many bacteria

Primary structure of α-amylase	No	No	In a few other bacterial genomes

Fatty Acid synthetase complex	No	Maybe	Exclusive to actinobacteria

Exospore formation	No	Yes	Exclusive to actinobacteria

HEH domain in Ku protein	No	No	Exclusive to actinobacteria

Many of these traits argue for an actinobacterial role in eukaryogenesis but not the origin of archaea. This list of informative characters is taken from [16].

**Table 6:** Summary of data that supports bacilli ancestry for archaea.

Trait	Supports Bacilli as ancestor of Neomura	Supports Bacilli as ancestor of the Archaea	Exclusive to Bacilli among Bacteria
Ribosomal S12 insert	Yes	Yes	Might be also be in Chloroflexi

Geranylgeranylglyceryl Phosphatase	No	Yes	Also in Bacteroides

Mevanolate pathway	Yes	Yes	In a few other bacterial genomes

Ancestors of n-linked glycoprotein	Yes	Yes	Closest hits to eukaryote genes are all from Bacilli

PyrD 1B	No	Yes	In Firmicutes and some Thermotogae

Two L7AE paralogs	Yes	Yes	Exclusive to Firmicutes

RecU	Yes	Yes	Exclusive to Firmicutes

The bacilli are more similar to archaea in terms of DNA repair, ribosome structure, and lipid metabolism than any other group of bacteria.

### Viruses as the missing link between the prokaryotic superkingdoms

Now that we have argued for the true distance between archaea and bacteria, the time has come to cross that desert. As we have asserted above, this is a unique event in evolution, so we must properly set the stage. The selective pressures associated with extreme environments and antibiotic warfare are ancient, however, they cannot cause a revolution on their own, so a significant relaxation in selective pressure is necessary. We argue that viral endosymbiosis could relax selective pressure enough to start such a revolution.

Koonin has observed that the PolB family of polymerases are the most common DNA polymerase in viruses [91]. Koonin *et al. *also observed that archaeo-eukaryotic DNA primase was a hallmark viral protein [19]. This hints at some connection in DNA replication between archaea, eukaryotes, and viruses. We examined the distribution of all protein families in Pfam [78] that originated at the root of archaea and eukaryotes to see if this connection could be extended. We defined Pfam familiess that were present in at least 90% of archaeal genomes (46 at the time) and 90% of eukaryotic genomes (35 at the time) and in less than 50% of bacterial genomes (939 at the time) as originating at the root of archaea and eukaryotes. A 90% cutoff is strict enough to imply that the protein was present in LAECA, while a 50% cutoff is loose enough to accommodate recent horizontal transfers. Most of these Pfam families are well below the 50% cutoff in bacteria.

By this definition there are 74 Pfam domains that originated in LAECA; 24 of these are found in at least one viral genome (Table 7). On average each of these Pfam domains is present in 36.38 viral genomes (14.36 if one excludes PolB). As an approximate measure of the significance of this result we took 10000 random samples of 74 Pfam domains that are found in at least one cellular genome to see how often one finds 24 or more in at least one viral genome. None of the random sets had that many viral Pfam domains, which implies this set is significantly enriched in viral proteins. However, we must keep in mind that our sampling of the viral world is still highly biased (discussed in [91]) and that viral genomes evolve rapidly. Viral genomes are sampled so poorly that none had the MCM domain from Pfam, even though it is found in a prophage region of some bacilli as discussed above. Further, 18 of the remaining Pfam proteins that originate in LAECA are ribosomal, which we assume are less advantageous for viruses to encode than the DNA replication machinery (although we did find several ribosomal proteins in viruses in this set).

**Table 7:** Pfam proteins that originated near LAECA and their distribution in the viral world.

PFAM ID	Description	Archaea	Bacteria	Eukaryotes	Viruses
PF00136	DNA polymerase family B	45	195	31	384
PF03104	DNA polymerase family B, exonuclease domain	45	210	31	173
PF01068	ATP dependent DNA ligase domain	45	372	31	50
PF01096	Transcription factor S-II (TFIIS)	45	1	31	44
PF00867	XPG I-region	43	0	31	37
PF04566	RNA polymerase Rpb2, domain 4	45	0	32	31
PF04567	RNA polymerase Rpb2, domain 5	45	0	32	31
PF01896	Eukaryotic and archaeal DNA primase small subunit	44	159	31	30
PF04675	DNA ligase N terminus	44	167	31	20
PF04679	ATP dependent DNA ligase C terminal region	44	248	31	20
PF00752	XPG N-terminal domain	43	0	31	16
PF00705	Proliferating cell nuclear antigen, N-terminal domain	45	0	33	10
PF01191	RNA polymerase Rpb5, C-terminal domain	44	0	32	5
PF00352	Transcription factor TFIID (or TATA-binding protein, TBP)	45	0	33	4
PF01194	RNA polymerases N/8 kDa subunit	45	0	30	3
PF01981	Peptidyl-tRNA hydrolase PTH2	45	26	30	3
PF00382	Transcription factor TFIIB repeat	45	0	32	3
PF03876	RNA polymerase Rpb7-like, N-terminal domain	42	0	31	2
PF08542	Replication factor C	44	0	31	2
PF02933	Cell division protein 48 (CDC48), domain 2	45	18	31	1
PF01599	Ribosomal protein S27a	41	0	32	1
PF02359	Cell division protein 48 (CDC48), N-terminal domain	45	44	32	1
PF01873	Domain found in IF2B/IF5	45	0	33	1
PF01253	Translation initiation factor SUI1	45	287	33	1

The Pfam familiess that originated in LAECA are more common in viruses than those that originated in LBCA.

We can also verify whether this result is significant by looking at the set of proteins that would be present in LBCA (last bacterial common ancestor), but not LEACA under the same definition, that is, Pfam domains present in at least 90% of bacterial genomes and less than 50% of archaeal and eukaryal genomes. There are 106 such Pfam domains and 15 of them are found in at least one viral genome (p-value 0.2457). Each of those 15 is in an average of 8.33 viral genomes. It should be noted that this is an underestimate for LBCA's content since there are so many parasitic bacteria with genomic sequences available. However, in general viruses share more Pfam domains with LAECA than LBCA.

Koonin proposes, based on PolB's distribution, that archaea arose from an acellular ancestor and then retained the more ancient polymerase [91]. We find this view hard to reconcile with the three independent arguments for the derived nature of archaea provided above. Forterre has argued that DNA originated from a viral endosymbiosis in each of the superkingdoms [17], but our data argues against that scenario for the origin of bacteria. We propose the alternative hypothesis that viral endosymbiosis occurred in bacteria and gave rise to archaea. This virus would supply the missing link in terms of DNA replication machinery between the prokaryotic superkingdoms. We think this would have to be endosymbiosis and not just a horizontal transfer given the distribution and interdependencies of these systems in cellular life.

To a first approximation there are three components that define the propensity of a genome to get permanently damaged. The first is the environment. Many different extreme environments are damaging to DNA, including radiation, high temperature and desiccation [92]. Second is the size of the genome. The larger the piece of DNA, the more likely damage will occur, and the more it must be mediated. Third is the state of the active repair system. If active repair is poor even rare damage events will eventually accumulate. Therefore, we argue that systems that are extreme in any one of these three components must routinely deal with DNA damage during replication.

Archaea, in general, fit the description of extremophile better than any other major taxa. It has been proposed that the unifying trait of all archaea is adaptation to chronic energy stress [93]. The author argues that archaea outcompete bacteria in niches that are under chronic stress. Thus archaea have become successful in dealing with environments that other superkingdoms cannot handle. The author noted that archaea do better in environments that are consistently extreme, and are outcompeted by bacteria in environments that fluctuate.

A corollary of chronic energy stress is chronic DNA damage. Many of the extremophilic environments archaea have made home severely damage DNA. On the other hand, bacteria may face occasional stressful situations and require DNA repair. Therefore it is disadvantageous for bacteria to have their repair systems on all the time. Conversely, archaea need to constantly repair their DNA, so it would make sense if the line is blurred between their replication and repair systems. An example of this prepare for the worst strategy is the unique ability of PolB to read ahead and stall replication if a uracil is encountered in archaea [94].

In terms of large genomes eukaryotes win hands down (see figure 1 in [95]). A polymerase is more likely to encounter damage somewhere in the replication of these large genomes than a prokaryote with a smaller genome in a similar environment. This is supported by evidence that eukaryotes use a separate repair system during replication of the large non-transcribed regions of their genome [96,97].

What other situation besides chronic DNA stress and large genome size would put similar pressure on the DNA replication machinery? We argue, somewhat counter intuitively, that a total lack of active DNA repair systems would create a similar situation. Again it is optimal for the replicative system to expect to encounter damage. Viruses fit that description perfectly as they are unable to actively maintain their genomes without their host.

If the repair systems were turned on more and more of the time, the main replicative system would become free to drift. Under this scenario the ancestors of archaea could mix and match bacterial repair and replication proteins with several molecular innovations and some transfers from the viral endosymbiont. The end result could be a system that is more robust to chronic stress. The canonical rooting implies that the components of the replication machinery that are homologous, but not orthologous, were independently recruited from proteins that initially processed RNA. Under either scenario the same amount of molecular innovation is required. The question then becomes, is it easier to innovate function in a RNA based organism or a DNA based organism under relaxed selective pressure? We argue that the difference cannot be quantified, as both scenarios predict exactly what we observe: some proteins are orthologs, some are homologs, and some are unrelated. Therefore the way to tell the difference between these scenarios is independent lines of evidence. The polarizations presented above imply the bacterial repair machinery was recruited to become the replication machinery of archaea.

It is also tempting to speculate that many of the features shared between viruses and the eukaryotic nucleus described in the viral eukaryogenesis hypothesis [73,98,99] could be extended to this hypothesis. Bell notes many similarities between nuclei and viral replication factories. One can imagine the ancestry of these traits going back to LAECA with some being lost in archaea, and others not developing until the root of eukaryotes. This is only consistent with our hypothesis if archaea are holophyletic, but for now it is certainly worth considering.

### The greatest battle ever fought

So far we have demonstrated that there is robust evidence that archaea are a derived superkingdom. We have shown the bacterial ribosome could have enough plasticity to evolve into an archaeal one. We have presented evidence that there is some link between DNA replication in archaea, eukaryotes and viruses that could be the result of endosymbiosis. Now we will try to combine these into the larger story of why a bacterium would evolve into an archaeon.

As we discussed above, we feel the greatest weakness of Gupta's invocation of antibiotics is it is not of sufficient evolutionary pressure to cause a revolution on the scale necessary to create the differences between the prokaryotic superkingdoms. Observations of the vast differences in DNA replication machinery and evidence of a viral endosymbiosis in a bacillus before LEACA will set the stage for our subsequent hypothesis.

In the traditional antibiotic battle the gram-positives are capable of evolving resistance to each other. This leads to what is commonly referred to as a Red Queen game [100]. Neither group ever really gets ahead in the long-term war as each defensive innovation is matched by an offensive one. But that does not mean there are never winners in battles on shorter time scales. Winning a battle is not a good thing in the long run. The winners will increase in population size and consume more of an environment's resources. The corollary is that they become a better target for less dominant species to kill. If a species evolves a more resistant ribosome it just puts more pressure on the rest of the community to hit other targets in that species.

One can imagine a firmicute deeply entrenched in such warfare endowed with the gift of a complete and novel replication system from a virus. This is supported by the distribution of viral Pfam proteins discussed above. The virosphere contains so much diversity that even rare combinations of genes would eventually end up in the same capsid at the same time as long as they have some advantage to any virus. It would be an incredibly rare event for the virus to be just right for the bacterium to take up the entire replication system. And thus the stage is partly set for why the revolution happened but once.

The core of the DNA replication system does not appear to be as common an antibiotic target as the ribosomes or RNA polymerase. A search of DrugBank revealed no antibiotics that target PolC [101]. However, there are several that target gyrase. Why the difference? Inhibition of PolC just stops a population from growing, but the damage induced by the loss of a functional gyrase invokes an SOS response and leads to cell death. There are probably natural antibiotics that target PolC, but they would not be as effective as the numerous ones that target the ribosome and RNA polymerase. Thus the introduction of PolB into the bacillus genome would not be the enough to start the revolution. This is supported by the fact that many proteobacteria use PolB as a repair enzyme, the result of a HGT that did not start a revolution.

As discussed above there are no bacteria that have archaeal histones. This strongly implies they are only compatible with the archaeal-eukaryal replication machinery. Thus we argue that viral endosymbiosis was a relaxation in selective pressure that in combination with pressure from antibiotics targeting gyrase led to the innovation of histones. This is not a trivial difference with Cavalier-Smith's hypothesis that the numerous differences between the DNA-handling machinery of bacteria and archaea are the result of histones dramatically changing the way in which this machinery could interact with DNA [16]. He argues this was an adaptation to thermophily.

However, Forterre has presented several arguments against Cavalier-Smith's scenario. He argues that the bacterial histone-like proteins that have replaced the archaeal ones in *Thermoplasma acidophilum *work just fine with the archaeal replication machinery [17]. He also notes that many hyperthermophilic bacteria do not use histones. At the same time hyperthermophilic bacteria exchange many genes with archaea [102]. Therefore the standard bacterial replication machinery could probably not tolerate the invention of histones even under selective pressure from an extreme environment. Euryarchaea appear to have gained DNA gyrase via several independent horizontal transfers from bacteria [47]. The fact that several euryarchaea retain both histones and gyrase is evidence against Cavalier-Smith's idea that gyrase became totally redundant with the advent of histones. That view is weakened further given that gyrase was found to be essential in several of those genomes [103].

Since pressure from thermophily alone could not force histone innovation, we invoke the viral endosymbiont hypothesis. In other bacteria an alternative system to gyrase would not be much of an advantage, as getting rid of gyrase would just put more pressure on targets like the ribosome and peptidoglycan synthesis. However, as discussed above, the bacilli have several unique ribosomal proteins. That means they could already have some adaptations and preadaptation to antibiotic warfare that makes them a difficult target to hit. As discussed above they have EpsE [104], which could preadapt them for functioning without peptidoglycan. Once gyrase was no longer a useful target they could quickly lose peptidoglycan in their cell walls. The loss of these two major targets would be a huge advantage and increase pressure on the ribosomes as a target.

At this point any change to the ribosome would be highly beneficial. One can imagine a Red Queen game where neomura have a distinct advantage over gram-positives but need constant innovation in their ribosomes to maintain that advantage. The observation that many archaeal-eukaryal ribosomal proteins bind Zn would be consistent with pressure to ensure proper assembly despite the antibiotics. This is supported by the fact that bacterial hyperthermophiles, whose environment interferes with ribosomal assembly, have more Zn binding sites than most other bacteria [35].

Thus the initial neomura would have an advantage in antibiotic warfare as well as the ability to replicate DNA even in the presence of damaging pressures. Their genomes could be much larger than extant prokaryotes. A large robust genome would allow neomuran to be oligotrophic and handle extreme environments. This would put them in direct competition with many bacteria in diverse environments. Their larger genome size would allow for more gene duplication, which could lead to structural innovations like the ribosomal proteins found in neomura but not bacteria.

The strongest support for this hypothesis comes from the antibiotic target site most studied in the ribosome: the 23S RNA between ribosomal proteins L22 and L4. L22 and L4 are conserved across teh superkingdoms. They bind to the same positions on the ribosome in all three superkingdoms. There are numerous crystal structures, from both prokaryotic superkingdoms, with antibiotics bound in these sites [105,106]. These studies demonstrated that nine different antibiotics that bind strongly to this site in bacteria bind with much less affinity in archaea. A2058 (*E. coli *numbering) is one of the sites on the 23S RNA directly involved in binding these drugs. A2058 is conserved across 99.4% of sequenced bacterial 23S rRNAs [107]. The site is almost universally guanine in archaea and eukaryotes. The mutation A2058G makes many bacteria macrolide-resistant [108], while the reverse mutation can make archaea macrolide-sensitive [109]. These differences in antibiotic affinity are well conserved across the divide between bacteria and neomura, and appear to be the result of intense selective pressure from antibiotics.

Even though bacteria are able to gain resistance through a similar mutation, it is probably not fixed because there is a slight decrease in fitness that can be reduced with other mutations [107]. If there were constant pressure on that site other mutations and changes in structure could relax those costs and fix that position. That would be completely consistent with the scenario outlined here. If the divide between archaea and bacteria is primordial, it is much harder to explain this difference. Ribosomal proteins L22 and L4 must have been present in LUCA. If the ancestor of archaea was an extremophile they should not have been in competition with enough bacteria to need the resistance inferred by this mutation.

It would be tempting to speculate that this mutation is an adaptation to thermophily or some other extreme environment to answer this nagging issue of antibiotic pressure at the root of archaea. Examining the position in bacterial hyperthermophiles can be tested. In both the hyperthermophiles *Aquifex aeolicus *and *Thermotoga maritime *this position is 100% conserved as adenine, as it is in their thermophilic relatives (Additional file 4; Figure S4). The thermophile *Thermus thermophilus *has two copies of the 23S RNA where usually both have adenine at that position unless they are under selective pressure from antibiotics [110]. Thus the only explanation that appears to hold water is some extreme antibiotic pressure at the root of archaea.

The mark of antibiotic pressure can also be seen in the proteins that would be lost at the origin of archaea. We searched Pfam and DrugBank for antibiotic targets that are conserved across bacteria but were clearly not in LACA. Eight of these are listed in Table 8. Several of these appear to have been horizontally transferred to archaea, such as DNA gyrase. That is consistent with the scenario under discussion because once archaea were no longer under strong antibiotic pressure these systems would be free to become essential again. It would be interesting to look at each of these eight predicted losses and see what preadaptations and environmental conditions can make them non-essential.

**Table 8:** Drug targets found across bacteria that were probably not in LACA.

Target	Example Drug
*Alanine Racemase*	Cycloserine

*Beta-lactamase*	Cefoxitin

Hsp90	Geldanamycin

*Cytochrome c*	Minocycline

*DNA gyrase*	Trovafloxacin

*3-oxoacyl-[acyl-carrier-protein] synthase 1*	Cerulenin

*Penicillin-binding proteins*	Cefoperazone

Peptidoglycan synthetase ftsI	Ertapenem

We argue these proteins were lost in the archaeal ancestor in response to a unique antibiotic warfare scenario. Targets in italics appear to have transferred to archaea after LACA.

**Table 9:** Examples of drugs targets sites with resistance in archaea.

Target Site	Example drugs
23s RNA between L22 and L4	Macrolides[106] and PTF inhibitors [105]

RNA Polymerase	GE23077[130]

EF-Tu	Kirromycin [131]

These drugs bind targets sites present in both bacteria and archaea (or eukaryotes), but with very different affinities. We argue this is a molecular fossil of the unique antibiotic war that resulted in the origin of archaea.

Why would this war end and who would the winners be? To address this question we will invoke the two novel niches that are central to the neomuran hypothesis; phagotrophy and hyperthermophily [16]. The oligotrophic neomuran with large genomes would be able to form many symbioses with prokaryotes because of their diverse metabolism. Such an environment would favor the preadapations to phagotrophy discussed in [111]. This could lead to several endosymbiotic events in a short span of time. These would force the nucleus to become a better separator in dealing with selective pressures proposed by several hypotheses: invasion of introns [112], differing metabolisms [113] and ribosome chimerism [114]. The successful phagotroph would eat prokaryotes, so at first it would be to the advantage of the prey to try to kill the neomura. However, that is not the optimal strategy for dealing with phagotrophs. It is much better to persist inside them and eat them from the inside out, as can be seen by the numerous bacterial taxa that have independently evolved the ability to infect eukaryotes. Once it is possible to infect the phagotrophs, killing them with antibiotics becomes counterproductive. And thus a truce (or new war) would be declared on one front of the great antibiotics war.

The early eukaryotes would outcompete and eat many of the initial neomura, but would be at a disadvantage in extreme environments as they began to rely on their cytoskeletons and larger cell size more. It would be easier for the neomura to drift into more extreme environments because of their DNA replication machinery. The proto-archaea would begin to emerge as the neomura began moving into previously unoccupied niches of extremophily. The conversion of their membranes would probably be the commitment step in the process. Once they began settling into environments that are constantly extreme they would be under pressure to streamline their genomes.

This scenario is consistent with a recent study on gene content evolution in archaea that concluded that most archaeal genomes have been streamlined from larger ancestral genomes [115]. The authors conclude that the archaeal ancestor could have had 2000 gene families, and the extant archaeal groups are mostly created through differential loss. The authors note this repeated loss is consistent with the energy shock state of the archaea described in [93], as specialization and loss are highly favorable in consistently extreme environments. The trend of euryarchaeal and crenarchaeal specific traits to both be present in the deep branching archaea is also consistent with the idea archaea became specialized from a more generalized genomic ancestor. The redundancy in archaeal systems such as two replicative polymerases and two cell division systems could be remnants of the antibiotic war. That redundancy would become unnecessary once archaea committed to extremophily. It was noted in [2] that ribosomal protein loss is much more common in archaea than in bacteria. Our hypothesis implies that the distribution of ribosomal proteins in archaea is the result of independent losses once they were no longer under antibiotic pressure. Some of these novel proteins developed other roles to deal with extremophily so they have been retained. The ancestral archaeal ribosome could very well have contained all of the proteins found in any archaeal genome, which would certainly weaken that aspect of the eocyte hypothesis.

**Figure 5:**
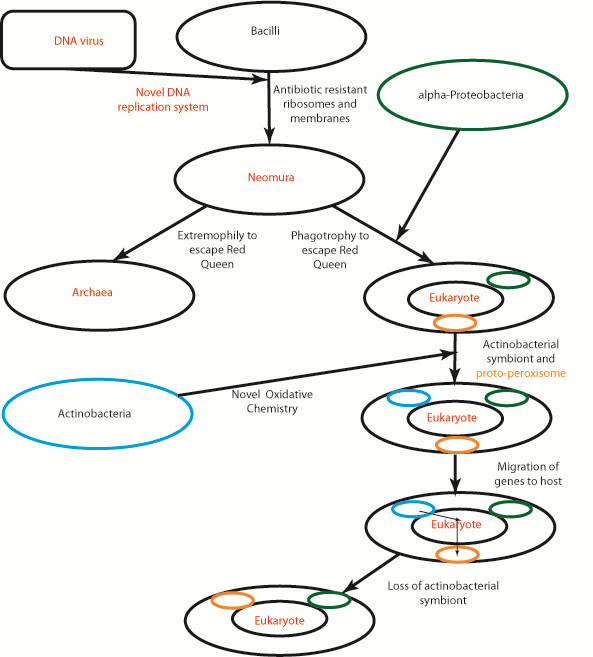
**Summary of our hypothesis**. A viral endosymbiosis bridges the gap in DNA machinery between the superkingdoms. That triggered an antibiotic war that resulted in the birth of eukaryotes and archaea. The antibiotic war ended when archaea became extremophiles and the eukaryotes became phagotrophs. Traits shared between eukaryotes and actinobacteria are the result of endosymbiosis; the peroxisome is not the direct descendent of an actinobacterium.

What about the neomura? They would be stuck in the middle. The eukaryotes would be eating them, and they would still be in competition with bacteria. Their only viable strategy would be constant innovation, as they would not really have a novel niche. However, the wave caused by viral endosybiosis would not go on forever. There would be diminishing returns in terms of the resistance provided by the new innovations. Eventually the innovations would become a disadvantage as bacteria can then release compounds that only target the new systems. For instance aphidicolin inhibits DNA replication in archaea and eukaryotes but not bacteria by targeting their unique polymerase [116,117]. So the initial advantage the neomura have in terms of antibiotic resistance is not a stable niche. They were outcompeted from three sides, and thus we are left with a hole in the middle of the branches of the tree of life that often gets mistaken as the root. This scenario is summarized in Figure 5.

## Discussion

It is reasonable to ask how different archaea and bacteria would have to be for us to consider the rooting debate closed. If the genetic material were different between the superkingdoms it would be strong evidence of life being polyphyletic. If the genetic codes were somewhat different (even a few codons), that would certainly be evidence that both groups were primordial. If membrane proteins like SecY were not universally conserved, we would take that as evidence LUCA was acellular. The differences between the prokaryotic superkingdoms seem small if we consider that the last prokaryotic common ancestor had a membrane and a ribosome that used the same genetic code as all extant life. They have more in common than can be described by any tree.

None of the differences between archaea and bacteria are great enough to imply a transition between the superkingdoms is impossible. The three independent polarizations provide compelling evidence the transition occurred. A viral endosymbiosis in a firmicute host could be the relaxation in selective pressure that acted in combination with pressure from antibiotics to cause a revolution in terms of membranes, ribosomes, and DNA replication machinery. This is supported by the association between proteins found in viral genomes and those that appear at the root of archaea and eukaryotes. Gupta's hypothesis that antibiotics led to the differences between the superkingdoms is well supported by the data generated in the past decade.

Archaea would certainly need to have several innovations in terms of protein structure. None of these are deal breakers. They are present in extant cells, and are not found in viruses. So they would have been an innovation at some point. There is no reason to assume all structural innovations happened near the root of the tree of life. Work from our group probing the relationship between ancient ocean chemistry and protein structure evolution is an example of one source of later innovations [36,37]. The modern ocean has several orders of magnitude more Zn than the ocean of LUCA's time [118]. Many Zn extant binding sites evolved after that transition. As noted above, several of the ribosomal proteins unique to the neomura have Zn binding sites. One of the innovations needed, PolD, is predicted to have two Zn fingers [39]. Increasing levels of Zn would not be the only factor, but it is another example of how the revolutionary planetary changes shape evolution as discussed in [119]. This observation makes sense if one places the origin of archaea after the great oxidation event, and considers the fossil record as a supplement to phylogenetic data. If we look at the details we may find the rhyme and reason to the other novel structures at the root of archaea as well. There are also many structural innovations at the root of the eukaryotes [120]. The fact that archaea have many unique protein structures does not imply they are primordial.

As we have hinted above, one of the strengths of this hypothesis is it does not rely on archaea being holophyletic. The scenario we have described implies holophyly, but if something conclusively proved archaea were ancestral to eukaryotes, it could be adapted. There is no explanation in the neomuran hypothesis for the traits shared between actinobacteria and eukaryotes besides vertical descent. The link Cavalier-Smith has justified could be the result of an endosymbiosis that did not leave its mark with an extant organelle. If archaea are paraphyletic it just means eukaryotes did not originate for the reasons we have hinted at here, but rather more along the lines of the traditional endosymbiotic hypotheses. It does not change the way we have to think about the origin and rooting of archaea, which is the central focus of this paper.

The hypothesis we have proposed can be refined with experiment. It seems if one really wants to understand the likelihood of intermediates between archaea and bacteria we need to understand why hybrid systems are unheard of. For instance, what other proteins need to be placed into a bacterium to allow them to use histones? How would an archaea with a bacterial ribosome function? Trying to recreate the intermediates we believe went extinct would certainly give insight into their plausibility. It definitely would give better insight into the functional nuances of proteins with homologous function across the prokaryotic superkingdoms that appear to be highly resistant to horizontal transfer. It would be highly informative regardless of the location of the root of the tree of life.

We have drawn our data from diverse sources that are not usually the primary tools for studying evolution. Viruses have been getting more attention as players in shaping the tree of life recently [18] and better sampling will clarify the plausibility of the endosymbiosis we have proposed. However, essentiality and protein structure are non-traditional tools in this field. If essentiality experiments were performed across the ribosomes and DNA replication machinery of bacilli under different conditions it could give us hints as to what selective pressures would need to be relaxed for the major transition to begin. Further study of natural antibiotics will also continue to increase the resolution of the hypothesis. We argue this line of experiment would be useful in its own right, since many of the firmicutes are pathogens that effect human health.

Of course indepth sampling of thaumarchaeota and korarchaeota is going to be invaluable to this endeavor. If the ribosomal proteins that are currently missing in these groups are found in new genomes it would imply independent losses and make holophyly seem a little more appealing. The redundancy left in these genomes could just be the first surprise. Deeper sampling may reveal redundancy in some of the archaeal-bacterial hybrid systems we discussed above. Finding a deep branching archaeon that uses a bacterial system would truly validate this hypothesis.

The proteasome is an important component of our hypothesis. If one roots archaea in bacilli it does not explain the presence of the proteasome across the entire superkingdom. We think Cavalier-Smith is correct in pointing it out as a link between archaea and actinobacteria, but in light of the other evidence raised here we do not find that argument convincing on its own. It is not clear what direction the proteasome was inherited. Even if the proteasome was horizontally transferred it does not weaken the polarization of archaea; it would still be a derived structure that was present at the root of archaea, so they must still be derived.

There are many instances in the literature where data are only presented under the canonical rooting, when in fact it is better explained by an alternative rooting. This quickly leads to circular logic; a hypothesis gets buried because no data supports it, data gets buried (in supplemental data or *ad hoc *invocations of HGT) because it does not fit with the canonical rooting. As an example look at how much data from Eugene Koonin's group we have cited in this work to support our hypothesis even though he has made it clear he thinks this rooting is unsupportable (see his reviews of [11,81] and this manuscript as well). We refute the view, and prevailing opinion, that there is no reasonable data to support a rooting within bacteria.

For instance, one of the biggest problems with the canonical rooting is the origin of cells. The term "RNA world" is sometimes invoked as a miracle that could explain anything that happened in evolution before cells look the way they do now. But one thing RNA definitely cannot do is to make transmembrane pores. This problem is addressed well by the obcell hypothesis of Blobel and Cavalier-Smith [121,122]. They propose proto-cells had very little going on inside them initially. Rather, they were collections of ribozymes tethered to the outside of a cell. The details of their proposal get around the problems of transmembrane RNA structures, but also implies the first true cell had a double membrane (like the gram-negative bacteria). Our point here is not about which hypothesis is correct; but that both of them are understood better in terms of the strengths and weaknesses of the other; throwing either out is essentially operating without a null hypothesis. The differences in these hypotheses was recently reviewed in [123].

Our view is that the debate should not be closed, but we acknowledge the difficulty in making meaningful contributions to that debate due to the complexity of the problem. Clearly DNA or protein sequence data alone does not suffice to provide a satisfactory answer. Data from different scales of biology - structure, function, biochemical processes, cell morphology etc. as well as the fossil record and earth's environment at different time points have to be applied. Fortunately, in our view, the increasing availability of these data and the tools to manipulate that data promise to keep the debate alive and opinion will continue to see-saw as it has done for the past 33 years since the pioneering work of Woese.

## Conclusions

This novel combination of hypotheses on the origin of archaea is intended to keep the debate alive. We think Cavalier-Smith has the best method for rooting the tree. His attention to detail and multiple sources of data allows one to refine his ideas as we have done here. Lake (and Gupta) has the right root for archaea, and despite our criticism, indel polarization is a useful methodology. Gupta has the right idea about antibiotics being a major force in this story and of course his work on indels laid the groundwork for our own work as well as Lake's. Forterre is right about viruses being major players in this event. Of course many others have shaped our thoughts on this subject, but we have clearly taken the most from the work of these four. In so doing we have tried to demonstrate the value of using opposing ideas as null hypotheses to each other.

Have we provided a scenario that explains every detail for how archaea evolved out of gram-positive bacteria? We certainly have not. What we have presented is a variety of data that attempts to show it is a plausible and defendable stance. The emergence of archea is an amazing event in the history of life, but decipherings its origin is not simple. However, if we close the debate we close our eyes to the large body of evidence that supports the polarization of this transition.

We have tried to provide a novel view on the origin of archaea that makes it clear very little is settled on this subject. We have provided a scenario that covers most of the transition between bacteria and archaea. The ideas we propose here can be refined with further experiment and more observations. The ideas are currently supported by diverse data. The study of these hypotheses will give us insight into several tangentially related topics that are worth pursuing such as the subtleties of antibiotic resistance in the ribosome in Gram-positives. In summary, the hypothesis we present and support here reconciles many opposing viewpoints and strongly argues that archaea are derived from Bacilli.

## Methods

Structural alignments were performed using CE [124]. Sequence alignments were performed using MUSCLE [125]. The alignments were visualized in Jalview [126]. Sequence trees were constructed using Phyml [127]. The essentiality of genes was determined by querying the database of essential of genes [31]. Drug targets were indentified from DrugBank [101]. The distribution of those targets was examined using Pfam [78].

## Competing interests

The authors declare that they have no competing interests.

## Authors' contributions

REV conceived the study and analyzed the data. PEB assisted in writing the manuscript. All authors read and approved the final manuscript.

## Reviewers' comments

### Reviewer's report 1

Patrick Forterre, Universite Paris Sud and Institut Pasteur, Paris, France

In their paper, « the origin of a derived superkingdom: how a gram positive bacterium crossed the desert to become an archaeon", Valas and Bourne update the previous proposal by Gupta linking Archaea to « gram positive bacteria ». The term gram positive bacteria is really outdated, since the work of Carl Woese has shown that it has no phylogenetic meaning. In fact, the title of this paper should be: "how a Firmicutes bacterium crossed the desert to become an archaeon ». Firmicutes are one of the 20, 30 more...(it's not yet clear) bacterial phyla. It has been much more extensively studied by human for medical and biotechnological reasons, but this does not qualify it to be more than that.

#### Author's response

*We find there are many compelling reasons to still consider the Gram-positives a monophyletic group as discussed in *[8]. *We have also presented evidence to justify why we do not trust the rRNA tree as a tool for macrophylogeny, especially for two groups nicknamed the "low gc gram-positives" and the "high gc gram-positives". We have two sources that disagree on the position of these groups. The solution is not to make declarative statements that one data source makes looking at other unreasonable, but rather to consider the strengths and weaknesses of each. One of the goals of the paper is to unify the hypotheses that question the rooting of the tree of life between the Achaea and Bacteria. Again, we'd like to point out that despite their differences Lake, Gupta, and Cavalier-Smith all agree the Archaea are derived from a Gram-positive bacterium. So even though we narrow it down to a phyla, we think this title still reflects the larger goal of the paper*.

In summary, Valas and Bourne proposed that both Archaea and Eukaryotes derived by transmutation from a member of Firmicutes, i.e. of one of the many bacterial phyla present today on our planet. This is revival of the old view that bacteria are primitive organisms that populated the planet much before all others (a sequel of Heckel monera). In fact, Bacteria are very evolved organisms, a superkingdom, that have been extremely successful sice they are now present everywhere and are usually much more abundant than members of the two other domains. It's unclear if they predated Archaea and ancient Eukarya, but they will certainly survive long after complex eukaryotes like us will have disappeared. I suspect that Archaea and Eukarya are the only two lineages that survive the extraordinary success of bacteria.

#### Author's response

*In this manuscript we have presented three pieces of evidence that imply Bacteria did predate the Archaea. This reviewer has not addressed why he feels those are insufficient*.

In my opinion, one of the reason for this success was the invention of DNA gyrase. This enzyme allows to couple directly the energetic state of the cell (the ATP/ADP ratio) to the expression of all genes at once in modulating the supercoiled state of the chromosome. Once you become addict to DNA gyrase, you can't let it go. The last bacterial common ancestor had a DNA gyrase, and all modern bacteria still have it. Some archaea succeeded to get gyrase from bacteria, they are now fully dependent of it. Plants also get gyrase from cyanobacteria, one possible reason for their success ?? The idea that a poor Firmicutes abandoned DNA gyrase to escape antigyrase drug producers does not seem realistic to me. Unfortunately for too many human patients, gyrases have found many way to become multi drug resistant without having to abandon it. In general, bacteria have been very efficient to thrive happily in all possible « deserts » that one can imagine, including hot springs up to 95°C. Hyperthermophilic bacteria or desiccation resistant bacteria are not en route to become archaea but bona fide bacteria. I cannot discuss in details all ad hoc hypotheses proposed by the authors to explain how a Firmicutes become an archaeon.

#### Author's response

*We argue that extreme habitats and antibiotic warfare were not unique enough niches in the manuscript, and this why do not think Gupta's or Cavalier-Smith's hypotheses are sufficient on their own. We agree that DNA gyrase is a big deal, and it would require some very unique circumstances for it to be lost. If the archaea are adapted to chronic energy stress it would not be unreasonable from them to move away from gyrase becuase the benefit you described above disappears if ATP is always scarce. The question is whether the transition is impossible or implausible. We are arguing this was a very rare event that only happened once. We feel it is more productive to try evaluating some of the hybrid systems we propose than to speculate about their impossibility. Once again, we regret a reviewer refuses to discuss details with us*.

They have certainly done a huge amont of bibliographic work and hard thinking which will help them in future debates on the origin of the three domains, but in my opinion, they have reached an impasse in trying to revive Gupta's hypothesis. For me, all hypotheses that invoke the transmutation of one domain (in its modern form) into another are definitively wrong. It is the same for hypotheses in which a combination of modern archaea and bacteria produced a protoeukaryote.

#### Author's response

*This implies there are essentially three primordial lineages; a view that we think is definitely wrong based on the currently available evidence. We have provided three robust pieces of evidence why the archaea appear younger than the bacteria which this reviewer has completely ignored. Other work from our group demonstrates that we can constrain the evolution of eukaryotes based on the biochemical history of the ocean *[36,37]; *that data argues the eukaryotes are a more recent lineage than the bacteria and archaea, and is completely independent of this work. Again this reviewer provides no argument for why transmutation hypotheses are definitely wrong, or why the polarization evidence bears no weight on these questions*.

I fully agree with Carl Woese who already wrote several years ago that « Modern cells are fully evolved entities. They are sufficiently complex, integrated, and "individualized" that further major change in their designs does not appear possible, which is not to say that relatively minor (but still functionally significant) variations on existing cellular themes cannot occur or that, under certain conditions, cellular design cannot degenerate". Firmicutes are modern cells, they cannot have experienced "major change in their designs" to become archaea and later on eukarya. These transmutation hypotheses put us backward in the pre-Woesian era, when evolution was viewed as a succession of steps from simple organism (moneraprokaryote-bacteria) to lower eukaryotes, then to higher eukaryotes, then to human (the scala natura). Definitely, a bacterium cannot be transmutated into an archaeon, even by a virus.

#### Author's response

*Only time will tell whether our skeptical reading of the rRNA tree will turn out to be pre-Woesian or post-Woesian. We do not express the view that evolution is just a series of successive steps or that a bacterial cell is simple in any way, and neither does Cavalier-Smith, Gupta, or Lake in our opinion. However within the larger process of evolution there are clear paths that were built in successive steps of increasing complexity. We think the best example of this is the proteasome's quaternary structure. Fortunately, the proteasome has an informative phylogenetic distribution that allows us to polarize the direction of its evolution. We argue the Archaea evolved from the Bacteria because their proteasome is more complicated, but that does do not imply the rest of the machinery is simpler in the Bacteria. If there are markers that are clear cut stories, how could using them for phylogenetic inference be pre-Woesian? Again, we ask why there are no polarizations that place the Bacteria as derived from the Archaea? We think the view that there is an insurmountable divide between the superkingdoms by definition to leads to circular reasoning, instead of a discussion about the actual data. Ironically, we see parallels between the current situation and Woese's account of how his ideas were first received *[5].

A virus take over of the replication apparatus could have created a bacterium with a novel replication apparatus, that's all. This would not have changed bacterial lipids, membranes, ribosomes, proteasomes, ATP synthases, transport sustems, metabolism,........ Possibly, one day, among the ten of thousand of bacteria whose genomes will be sequences, one will find one bacterium with an atypical replication system of recent viral origin, but I bet that this bacterium will have « bacterial ribosomes » and so on.

#### Author's response

*We would be very surprised if the DNA replication system was that different and rest of the cell was purely bacterial. The nice thing is this is one of the few points we disagree on that data will actually make clearer*.

If one want to understand the origin of modern domains, one has to consider that they originated in a very different world that our present one. A world with many lineages (domains or protodomains) that have now disappeared, possibly back to the cellular RNA world. This is a really difficult and fascinating objective which requires to propose sometimes bold hypotheses, but these hypotheses should take into account that the divide between the three modern domains is now so great that it cannot be crossed, even by an adventurous, desperate Firmicutes.

#### Author's response

*We again refer readers to work from our group on the evolution of the superkingdoms in relationship to history of ocean's biochemistry *[36,37]. *We will continue to incorporate new data sources that allow us to measure how different that world was instead of speculating about it. For now, the many data sources we have woven together imply there is something deeply wrong with the canonical rooting as well the logic used to support it (see reviewer #3's comments). This reviewer's advice that we need bold hypotheses, but the rooting must be taken as dogma makes little sense to us in light of the many problems with that rooting*.

### Reviewer's report 2

Eugene V Koonin, National Center for Biotechnology Information, NIH, Bethesda, Maryland, United States

To this reviewer, the manuscript by Valas and Bourne is frustrating. These authors continue to question the primary divide in the evolution of cellular life, that between archaea and bacteria, without any legitimate grounds. Here they go deeper into this falsehood by trying to present arguments for one bacterial root of archaea as opposed to another that has been proposed by a different author, in an equally faulty manner. Another innovation here is adding insult to injury: "This data have been dismissed because those who support the canonical rooting between the prokaryotic superkingdoms cannot imagine how the vast divide between the prokaryotic superkingdoms could be crossed." This allegation is a substantial part of the exceptionally brief abstract of Valas and Bourne. No comment seems to be required.

My general view which I see no reason whatsoever to change is expressed in the following quote from my review of a previous publication by the same authors:

"The nature of the primary divide in prokaryotes - and actually among all cellular life forms is clear, and it is between archaea and bacteria. This view is supported by the fundamental differences between archaeal and bacterial systems of DNA replication, core transcription, translation, and membrane biogenesis - essentially, all central cellular systems (not just the replication system as noted in the present paper). I believe these differences are sufficient to close the "root debate" (regardless of the appropriateness or lack thereof of the very notion of a root in this context) and to base analyses and discussions aimed at the elucidation of the nature of LUCA on that foundation." [11]

Perhaps, it is worth adding the results of a recent comprehensive analysis of phylogenetic trees for prokaryotic proteins that firmly supports the primary divide between archaea and bacteria [128].

#### Author's response

*A large part of the motivation for this manuscript was the review of our previous work *[11]. *Cleary there are others besides us who do not find things as clear cut as this reviewer (see reviewer #3 comments). We think there many reasons to support the canonical rooting, as well as reasons to questions it. We have presented our views on much of this evidence. The reviewer has again refused to discuss our data in any detail implying it is obvious why we are wrong. We feel we have greatly strengthened our previous argument by looking at the big picture in terms of the Gram-negative rooting. This reviewer claimed that rooting was unsupportable because it is so obvious that Achaea did not evolve from Bacteria. We feel we have strengthened that view, but clearly we have not swayed this reviewer. We do not think there is anything more to say on this subject so we point readers to the discussion between this reviewer and Cavalier-Smith in *[81].

The only other comment I wish to make is the extreme carelessness with which the manuscript is written. The abstract consists of 6 sentences of which two are obviously ungrammatical. Furthermore, in the Conclusion section of the abstract, the astonished reader finds "antibiotic warfare and a viral endosymbiosis" for which no argument and no mention has been made in the Results section. Perhaps, the authors can get rid of these and other similar problems in a revision but I do want to keep it in the record that this is how the manuscript was submitted for review.

#### Author's response

*We apologize for any issues with the form that took away from the content, and we hope the final version is improved. The paper is written somewhat recklessly because it is what it is: the end of a Ph.D. dissertation. We feel it was the right time to get these ideas out because of our perception that the canonical rooting is too dogmatic. This review has only supported our view this manuscript was needed. We think a reader should be astonished by the end of a short abstract before a long reckless paper; it gets them to read paper*.

*We find it interesting that this reviewer had no comment on two aspects of this work which can be judged independently of the rooting issue; the holophyly of the archaea and the actinobacteria's role in eukaryogenesis. We have presented much evidence that the conclusion this reviewer reached on the former, using indel data, is flawed. It would have been informative to hear this reviewer's opinion on that analysis*.

### Reviewer's report 3

Gaspar Jekely, European Molecular Biology Laboratory, Heidelberg, Germany

In this paper Vales and Bourne address a very difficult problem in evolutionary cellbiology, that of the origin of Archaea (archaebacteria). They do this after arguing at length for the bacterial rooting of the tree of life. Such attempts are very welcome, since these areas are extremely controversial and important, yet few people seem to notice that there is a problem there, namely that the conventional rooting of the tree of life between archaea and bacteria is far from being proven and as trivial as it seems. The evidence for this rooting, coming from paralogous gene rootings is highly questionable, and givesconflicting results when different paralogs are analyzed.

#### Author's response

*We thank this reviewer for demonstrating that not everyone thinks our line of questioning is as unreasonable as reviewers 1 and 2. There are certainly problems with any rooting, and the question is still very open at this point in our minds. Many experts share the opinions of those reviewers, but we feel these points are often swept under the rug when invoking that rooting*.

Notwithstanding these problems, conventional wisdom holds, as the authors rightly point out, that the position of the root is between the two domains (superphyla) bacteria and archaea, since these are the groups that are most distinct from each other. However, such rooting based on maximum divergence can often be wrong, since an ancestral group can give rise to highly derived groups. I don't have particular problems with uprooting the tree of life and abandoning the conventional rooting, but I find the evidence, as presented in this paper, quite week. I also have the feeling that the three indels and the distribution of the proteosome will not convince too many people to favour one rooting over the other. In all cases one can conceive scenarios that are in agreement with the conventional rooting, such as the presence of both forms in the last common ancestor and then differential losses in the stem bacterial and archaeal lineage. I acknowledge that some of this would be less parsimonious than under a bacterial rooting, but given that there are only very few characters that can be used, such less parsimonious scenarios can still easily be defended.

#### Author's response

*The strength of our evidence rests in the difference between polarization and parsimony which we have expressed before *[11]*:*

"To us parsimony can be used to analyze events where gain and loss have nearly equal probabilities, while polarizations imply that one direction would evolve more easily than the other. Consider the example of the proteasome discussed in detail in Cavalier-Smith 2002. A parsimony argument would be that the 20s proteasome is the result of a duplication so a non duplicated structure must precede it. The polarization argument involves considering the structure and function of proteasomes as well as the fitness of the intermediates to argue that evolution towards the 20s proteasome is much more plausible than the reverse direction. There are probably many cases where evolution has not been parsimonious, and we do not think parsimony is a safe or productive assumption. However, there appears to be many polarizable transitions and hopefully there are many more waiting to be discovered. "

*We do not think the polarizations make this an open and shut case. However, we find they are sufficient to question the canonical rooting and search for more evidence to support the alternative rooting we support here*.

The rooting issue aside, my main concern is that the scenario for the origin of archaea is not worked out well enough at the moment, and this contrasts with the length and ambition of the paper. The authors invoke an endosymbiosis with a virus to explain the origin of the archaeal DNA-handling enzymes. What does this exactly mean? Is it a lyzogenic virus? The term viral endosymbiosis does not seem to be the best choice here. The authors then invoke a very improbably form of infection with a virus that had collected from the virosphere the right combination of genes, to hand it over to the cell. In this way they try create a unique event that led to the unique origin of the archaea. I am not sure that invoking such a hypothetical, extremely rare event, for which there is no evidence, solves the problem. I acknowledge the enrichment of proteins of possible viral origin in the stem archaea, but this slight statistical enrichment does not mean that there was only one virus infection involved. One could just as well imagine a series of viral gene transfers in the framework of the antibiotic warfare scenario that provided the novel enzymes in a step-by step manner. Given the random sequence of events and the nature of the transferred genes, this could also lead to a lineage with unique identity. This scenario is at present the weakest part of the paper and should be worked out much better in a more focused paper.

#### Author's response

*We completely agree with this assessment of our viral endosymbiotic scenario. Endosymbiosis is probably not the best term, but we want to stress the radical nature of this interaction. We do not think our assumption about a rare virus is off base. The virosphere is very large and viruses are experts at manipulating genetic material in novel ways. The DNA handling enzymes did evolve twice despite how unlikely it was. We think a radical turnover of the machinery is only possible in a virus. Successive viral transfers could explain the data too, but this would still be a rare event to account for so many genes. An event like this would not leave much of mark besides a statistical enrichment (if even that). If that enrichment is real the question becomes why do the Archaea interact differently with viruses than Bacteria? That answer cannot be developed too much more at present, but a better sampling of the virosphere is definitely going to help here*.

There are several other ideas in the paper that are potentially interesting, but not well worked out. The proposition of an actinobacterial symbiont during early eukaryote evolution is one example. Such a hypothesis could possibly be spelled out in a full paper, with a detailed scenario and all the evidence that seems to support such a model. In this form it is just a proposition that is hard to judge thoroughly, and is very easy to dismiss. In general, my recommendation would be to refocus the paper around one key idea, namely the origin of archaeal DNA-handling enzymes by quantum evolution and from viral sources as a result of an antibiotics arms race. If the authors spell out this scenario clearly, together with the supporting evidence, but without going into the details of the rooting issue (discussed already in their previous Biology Direct paper) and the origin of eukaryotes (this could be done in a separate paper), this could become a much more useful and potentially influential manuscript. The title could then be changed accordingly. In the present form the title gives the impression that the authors wish to explain everything, which is far from being the case (for example the unique membrane chemistry or archaeal flagella are not covered).

#### Author's response

*This is a fair assessment of the manuscript. It is certainly overly ambitious and in many ways incomplete. The goal of this paper was to unite the various ideas about bacterial rootings of the Archaea. The fact that many of our ideas could be developed further is more evidence this debate is not as closed as the two other reviewers have declared. While we certainly have omitted many details, we have chosen to take a big picture view. There is an obvious connection between the problems rooting of the tree of life and the origin of the superkingdoms. We think we can only judge a rooting hypothesis by assessing how well it addresses these questions. We think the canonical rooting is insufficient when one begins asking questions on the origins of the superkingdoms. We hope that readers will pick and choose which ideas they like and continue to develop and test them*.

## Supplementary Material

Additional file 1 **Supplemental Figure 1. Alignment of RadA sequences from representative archaea**. Click here for file

Additional file 2 **Supplemental Figure 2. Maximum likelihood tree of GatD argues for multiple horizontal transfers**. This tree is not well resolved, but it does not support archaeal ancestry for eukaryotic proteins. Euryarchaeal sequences are highlighted in green, crenarchaea are magenta, thaumarchaeota are cyan, and korarchaeota are blue. The region of the indel is highlighted in red. There is no informative indel in this gene as was initially reported. Click here for file

Additional file 3 **Supplemental Figure 3. Sequence alignment of EF-1 from representative archaea**. Euryarchaeal sequences are highlighted in green, crenarchaea are magenta, thaumarchaea are cyan, and korarchaeota are blue. The region of the indel is highlighted in red. This alignment implies several reversions. Therefore this indel is not robust enough to determine whether archaea are holophyletic or paraphyletic. Click here for file

Additional file 4 **Supplemental Figure 4. 23s rRNA A2058 (*E. coli *numbering) is well conserved across bacterial hyperthermophiles**. This implies the conserved guanine in that position in archaea is not an adaptation to thermophily. Click here for file

## References

[B1] Woese CR Fox GE Phylogenetic structure of the prokaryotic domain: the primary kingdoms Proc Natl Acad Sci USA 1977 74 5088 5090 10.1073/pnas.74.11.5088 270744 PMC432104

[B2] Lecompte O Ripp R Thierry JC Moras D Poch O Comparative analysis of ribosomal proteins in complete genomes: an example of reductive evolution at the domain scale Nucleic Acids Res 2002 30 5382 5390 10.1093/nar/gkf693 12490706 PMC140077

[B3] De Rosa M Gambacorta A Gliozzi A Structure, biosynthesis, and physicochemical properties of archaebacterial lipids Microbiol Rev 1986 50 70 80 3083222 10.1128/mr.50.1.70-80.1986 PMC373054

[B4] Leipe DD Aravind L Koonin EV Did DNA replication evolve twice independently? Nucleic Acids Res 1999 27 3389 3401 10.1093/nar/27.17.3389 10446225 PMC148579

[B5] Woese CR The Archaeal Concept and the World it Lives in: A Retrospective Photosynth Res 2004 80 361 372 10.1023/B:PRES.0000030445.04503.e6 16328833

[B6] Koonin EV Martin W On the origin of genomes and cells within inorganic compartments Trends Genet 2005 21 647 654 10.1016/j.tig.2005.09.006 16223546 PMC7172762

[B7] Battistuzzi FU Hedges SB A major clade of prokaryotes with ancient adaptations to life on land Mol Biol Evol 2009 26 335 343 10.1093/molbev/msn247 18988685

[B8] Cavalier-Smith T Rooting the tree of life by transition analyses Biol Direct 2006 1 19 10.1186/1745-6150-1-19 16834776 PMC1586193

[B9] Gupta RS Protein phylogenies and signature sequences: A reappraisal of evolutionary relationships among archaebacteria, eubacteria, and eukaryotes Microbiol Mol Biol Rev 1998 62 1435 1491 9841678 10.1128/mmbr.62.4.1435-1491.1998 PMC98952

[B10] Lake JA Servin JA Herbold CW Skophammer RG Evidence for a new root of the tree of life Syst Biol 2008 57 835 843 10.1080/10635150802555933 19085327

[B11] Valas RE Bourne PE Structural analysis of polarizing indels: an emerging consensus on the root of the tree of life Biol Direct 2009 4 30 10.1186/1745-6150-4-30 19706177 PMC3224940

[B12] Skophammer RG Servin JA Herbold CW Lake JA Evidence for a gram-positive, eubacterial root of the tree of life Mol Biol Evol 2007 24 1761 1768 10.1093/molbev/msm096 17513883

[B13] Lake JA Henderson E Oakes M Clark MW Eocytes: a new ribosome structure indicates a kingdom with a close relationship to eukaryotes Proc Natl Acad Sci USA 1984 81 3786 3790 10.1073/pnas.81.12.3786 6587394 PMC345305

[B14] Rivera MC Lake JA Evidence that eukaryotes and eocyte prokaryotes are immediate relatives Science 1992 257 74 76 10.1126/science.1621096 1621096

[B15] Cavalier-Smith T The phagotrophic origin of eukaryotes and phylogenetic classification of Protozoa Int J Syst Evol Microbiol 2002 52 297 354 11931142 10.1099/00207713-52-2-297

[B16] Cavalier-Smith T The neomuran origin of archaebacteria, the negibacterial root of the universal tree and bacterial megaclassification Int J Syst Evol Microbiol 2002 52 7 76 11837318 10.1099/00207713-52-1-7

[B17] Forterre P Three RNA cells for ribosomal lineages and three DNA viruses to replicate their genomes: a hypothesis for the origin of cellular domain Proc Natl Acad Sci USA 2006 103 3669 3674 10.1073/pnas.0510333103 16505372 PMC1450140

[B18] Forterre P The origin of viruses and their possible roles in major evolutionary transitions Virus Res 2006 117 5 16 10.1016/j.virusres.2006.01.010 16476498

[B19] Koonin EV Senkevich TG Dolja VV The ancient Virus World and evolution of cells Biol Direct 2006 1 29 10.1186/1745-6150-1-29 16984643 PMC1594570

[B20] McGeoch AT Bell SD Extra-chromosomal elements and the evolution of cellular DNA replication machineries Nat Rev Mol Cell Biol 2008 9 569 574 10.1038/nrm2426 18523437

[B21] Boucher Y Kamekura M Doolittle WF Origins and evolution of isoprenoid lipid biosynthesis in archaea Mol Microbiol 2004 52 515 527 10.1111/j.1365-2958.2004.03992.x 15066037

[B22] Wong JT Chen J Mat WK Ng SK Xue H Polyphasic evidence delineating the root of life and roots of biological domains Gene 2007 403 39 52 10.1016/j.gene.2007.07.032 17884304

[B23] Iyer LM Abhiman S Maxwell Burroughs A Aravind L Amidoligases with ATP-grasp, glutamine synthetase-like and acetyltransferase-like domains: synthesis of novel metabolites and peptide modifications of proteins Mol Biosyst 2009 5 1636 1660 10.1039/b917682a 20023723 PMC3268129

[B24] Valas RE Bourne PE Rethinking proteasome evolution: two novel bacterial proteasomes J Mol Evol 2008 66 494 504 10.1007/s00239-008-9075-7 18389302 PMC3235984

[B25] Skophammer RG Herbold CW Rivera MC Servin JA Lake JA Evidence that the root of the tree of life is not within the Archaea Mol Biol Evol 2006 23 1648 1651 10.1093/molbev/msl046 16801395

[B26] Klein DJ Moore PB Steitz TA The roles of ribosomal proteins in the structure assembly, and evolution of the large ribosomal subunit J Mol Biol 2004 340 141 177 10.1016/j.jmb.2004.03.076 15184028

[B27] Harms J Schluenzen F Zarivach R Bashan A Gat S Agmon I Bartels H Franceschi F Yonath A High resolution structure of the large ribosomal subunit from a mesophilic eubacterium Cell 2001 107 679 688 10.1016/S0092-8674(01)00546-3 11733066

[B28] O'Brien TW Properties of human mitochondrial ribosomes IUBMB Life 2003 55 505 513 14658756 10.1080/15216540310001626610

[B29] Ciccarelli FD Doerks T von Mering C Creevey CJ Snel B Bork P Toward automatic reconstruction of a highly resolved tree of life Science 2006 311 1283 1287 10.1126/science.1123061 16513982

[B30] Cox CJ Foster PG Hirt RP Harris SR Embley TM The archaebacterial origin of eukaryotes Proc Natl Acad Sci USA 2008 105 20356 20361 10.1073/pnas.0810647105 19073919 PMC2629343

[B31] Zhang R Ou HY Zhang CT DEG: a database of essential genes Nucleic Acids Res 2004 32 D271 272 10.1093/nar/gkh024 14681410 PMC308758

[B32] Fang G Rocha E Danchin A How essential are nonessential genes? Mol Biol Evol 2005 22 2147 2156 10.1093/molbev/msi211 16014871

[B33] Drummond DA Wilke CO Mistranslation-induced protein misfolding as a dominant constraint on coding-sequence evolution Cell 2008 134 341 352 10.1016/j.cell.2008.05.042 18662548 PMC2696314

[B34] Sutter M Striebel F Damberger FF Allain FH Weber-Ban E A distinct structural region of the prokaryotic ubiquitin-like protein (Pup) is recognized by the N-terminal domain of the proteasomal ATPase Mpa FEBS Lett 2009 583 3151 3157 10.1016/j.febslet.2009.09.020 19761766

[B35] Makarova KS Ponomarev VA Koonin EV Two C or not two C: recurrent disruption of Zn-ribbons, gene duplication, lineage-specific gene loss, and horizontal gene transfer in evolution of bacterial ribosomal proteins Genome Biol 2001 2 RESEARCH 0033 11574053 10.1186/gb-2001-2-9-research0033 PMC56895

[B36] Dupont CL Yang S Palenik B Bourne PE Modern proteomes contain putative imprints of ancient shifts in trace metal geochemistry Proc Natl Acad Sci USA 2006 103 17822 17827 10.1073/pnas.0605798103 17098870 PMC1635651

[B37] Dupont CL Butcher A Valas RE Bourne PE Caetano-Anolles G History of biological metal utilization inferred through phylogenomic analysis of protein structures Proc Natl Acad Sci USA 2010 107 10567 10572 10.1073/pnas.0912491107 20498051 PMC2890839

[B38] Filee J Forterre P Sen-Lin T Laurent J Evolution of DNA polymerase families: evidences for multiple gene exchange between cellular and viral proteins J Mol Evol 2002 54 763 773 10.1007/s00239-001-0078-x 12029358

[B39] Tahirov TH Makarova KS Rogozin IB Pavlov YI Koonin EV Evolution of DNA polymerases: an inactivated polymerase-exonuclease module in Pol epsilon and a chimeric origin of eukaryotic polymerases from two classes of archaeal ancestors Biol Direct 2009 4 11 10.1186/1745-6150-4-11 19296856 PMC2669801

[B40] Koonin EV Wolf YI Kondrashov AS Aravind L Bacterial homologs of the small subunit of eukaryotic DNA primase J Mol Microbiol Biotechnol 2000 2 509 512 11075926

[B41] Della M Palmbos PL Tseng HM Tonkin LM Daley JM Topper LM Pitcher RS Tomkinson AE Wilson TE Doherty AJ Mycobacterial Ku and ligase proteins constitute a two-component NHEJ repair machine Science 2004 306 683 685 10.1126/science.1099824 15499016

[B42] Yutin N Koonin EV Evolution of DNA ligases of Nucleo-Cytoplasmic Large DNA viruses of eukaryotes: a case of hidden complexity Biol Direct 2009 4 51 10.1186/1745-6150-4-51 20021668 PMC2806865

[B43] Samuels M Gulati G Shin JH Opara R McSweeney E Sekedat M Long S Kelman Z Jeruzalmi D A biochemically active MCM-like helicase in Bacillus cereus Nucleic Acids Res 2009 37 4441 4452 10.1093/nar/gkp376 19474351 PMC2715239

[B44] Ammelburg M Frickey T Lupas AN Classification of AAA+ proteins J Struct Biol 2006 156 2 11 16828312 10.1016/j.jsb.2006.05.002

[B45] Chen YJ Yu X Egelman EH The hexameric ring structure of the Escherichia coli RuvB branch migration protein J Mol Biol 2002 319 587 591 10.1016/S0022-2836(02)00353-4 12054856

[B46] Komori K Sakae S Shinagawa H Morikawa K Ishino Y A Holliday junction resolvase from Pyrococcus furiosus: functional similarity to Escherichia coli RuvC provides evidence for conserved mechanism of homologous recombination in Bacteria, Eukarya, and Archaea Proc Natl Acad Sci USA 1999 96 8873 8878 10.1073/pnas.96.16.8873 10430863 PMC17700

[B47] Forterre P Gribaldo S Gadelle D Serre MC Origin and evolution of DNA topoisomerases Biochimie 2007 89 427 446 10.1016/j.biochi.2006.12.009 17293019

[B48] Forterre P Gadelle D Phylogenomics of DNA topoisomerases: their origin and putative roles in the emergence of modern organisms Nucleic Acids Res 2009 37 679 692 10.1093/nar/gkp032 19208647 PMC2647321

[B49] Becerra A Delaye L Islas S Lazcano A The Very Early Stages of Biological Evolution and the Nature of the Last Common Ancestor of the Three Major Cell Domains Annual Review of Ecology, Evolution, and Systematics 2007 38 361 379 10.1146/annurev.ecolsys.38.091206.095825

[B50] Yutin N Makarova KS Mekhedov SL Wolf YI Koonin EV The deep archaeal roots of eukaryotes Mol Biol Evol 2008 25 1619 1630 10.1093/molbev/msn108 18463089 PMC2464739

[B51] Brochier-Armanet C Boussau B Gribaldo S Forterre P Mesophilic Crenarchaeota: proposal for a third archaeal phylum, the Thaumarchaeota Nat Rev Microbiol 2008 6 245 252 10.1038/nrmicro1852 18274537

[B52] Elkins JG Podar M Graham DE Makarova KS Wolf Y Randau L Hedlund BP Brochier-Armanet C Kunin V Anderson I A korarchaeal genome reveals insights into the evolution of the Archaea Proc Natl Acad Sci USA 2008 105 8102 8107 10.1073/pnas.0801980105 18535141 PMC2430366

[B53] Martin W Muller M The hydrogen hypothesis for the first eukaryote Nature 1998 392 37 41 10.1038/32096 9510246

[B54] Ettema TJ Bernander R Cell division and the ESCRT complex: A surprise from the archaea Commun Integr Biol 2009 2 86 88 19704896 10.4161/cib.7523 PMC2686351

[B55] Altschul SF Gish W Miller W Myers EW Lipman DJ Basic local alignment search tool J Mol Biol 1990 215 403 410 2231712 10.1016/S0022-2836(05)80360-2

[B56] Schmitt E Panvert M Blanquet S Mechulam Y Structural basis for tRNA-dependent amidotransferase function Structure 2005 13 1421 1433 10.1016/j.str.2005.06.016 16216574

[B57] Schwartz JH Reeves JY Broome JD Two L-asparaginases from E. coli and their action against tumors Proc Natl Acad Sci USA 1966 56 1516 1519 10.1073/pnas.56.5.1516 5339624 PMC220017

[B58] Poole AM Penny D Evaluating hypotheses for the origin of eukaryotes Bioessays 2007 29 74 84 10.1002/bies.20516 17187354

[B59] Hou S Makarova KS Saw JH Senin P Ly BV Zhou Z Ren Y Wang J Galperin MY Omelchenko MV Complete genome sequence of the extremely acidophilic methanotroph isolate V4, Methylacidiphilum infernorum, a representative of the bacterial phylum Verrucomicrobia Biol Direct 2008 3 26 10.1186/1745-6150-3-26 18593465 PMC2474590

[B60] De Mot R Actinomycete-like proteasomes in a Gram-negative bacterium Trends Microbiol 2007 15 335 338 10.1016/j.tim.2007.06.002 17587582

[B61] Klimke W Agarwala R Badretdin A Chetvernin S Ciufo S Fedorov B Kiryutin B O'Neill K Resch W Resenchuk S The National Center for Biotechnology Information's Protein Clusters Database Nucleic Acids Res 2009 37 D216 223 10.1093/nar/gkn734 18940865 PMC2686591

[B62] Wilson D Madera M Vogel C Chothia C Gough J The SUPERFAMILY database in 2007: families and functions Nucleic Acids Res 2007 35 D308 313 10.1093/nar/gkl910 17098927 PMC1669749

[B63] Nes WR Nes WD Lipids in evolution 1980 New York: Plenum Press

[B64] Lamb DC Kelly DE Manning NJ Kelly SL A sterol biosynthetic pathway in Mycobacterium FEBS Lett 1998 437 142 144 10.1016/S0014-5793(98)01218-6 9804188

[B65] Desmond E Gribaldo S Phylogenomics of Sterol Synthesis: Insights into the Origin, Evolution, and Diversity of a Key Eukaryotic Feature Genome Biol Evol 2009 2009 364 381 10.1093/gbe/evp036 PMC2817430 20333205

[B66] Smucker RA Ortiz-Ortiz L, Bojalil LF, Yakoleff V Biochemistry of the *Streptomyces *spore sheath Biological, biochemical, and biomedical aspects of actinomycetes 1984 xv Orlando: Academic Press 643

[B67] Wehenkel A Bellinzoni M Grana M Duran R Villarino A Fernandez P Andre-Leroux G England P Takiff H Cervenansky C Mycobacterial Ser/Thr protein kinases and phosphatases: physiological roles and therapeutic potential Biochim Biophys Acta 2008 1784 193 202 17869195 10.1016/j.bbapap.2007.08.006

[B68] Macek B Mijakovic I Olsen JV Gnad F Kumar C Jensen PR Mann M The serine/threonine/tyrosine phosphoproteome of the model bacterium Bacillus subtilis Mol Cell Proteomics 2007 6 697 707 10.1074/mcp.M600464-MCP200 17218307

[B69] Sandoval-Calderon M Geiger O Guan Z Barona-Gomez F Sohlenkamp C A eukaryote-like cardiolipin synthase is present in Streptomyces coelicolor and in most actinobacteria J Biol Chem 2009 284 17383 17390 10.1074/jbc.M109.006072 19439403 PMC2719378

[B70] Da Lage JL Feller G Janecek S Horizontal gene transfer from Eukarya to bacteria and domain shuffling: the alpha-amylase model Cell Mol Life Sci 2004 61 97 109 10.1007/s00018-003-3334-y 14704857 PMC11138599

[B71] Leibundgut M Maier T Jenni S Ban N The multienzyme architecture of eukaryotic fatty acid synthases Curr Opin Struct Biol 2008 18 714 725 10.1016/j.sbi.2008.09.008 18948193

[B72] Cavalier-Smith T The origin of eukaryotic and archaebacterial cells Ann N Y Acad Sci 1987 503 17 54 10.1111/j.1749-6632.1987.tb40596.x 3113314

[B73] Bell PJ Sex and the eukaryotic cell cycle is consistent with a viral ancestry for the eukaryotic nucleus J Theor Biol 2006 243 54 63 10.1016/j.jtbi.2006.05.015 16846615

[B74] Aravind L Koonin EV Prokaryotic homologs of the eukaryotic DNA-end-binding protein Ku, novel domains in the Ku protein and prediction of a prokaryotic double-strand break repair system Genome Res 2001 11 1365 1374 10.1101/gr.181001 11483577 PMC311082

[B75] Grangeasse C Cozzone AJ Deutscher J Mijakovic I Tyrosine phosphorylation: an emerging regulatory device of bacterial physiology Trends Biochem Sci 2007 32 86 94 10.1016/j.tibs.2006.12.004 17208443

[B76] Kanehisa M The KEGG database Novartis Found Symp 2002 247 91 101 discussion 101-103, 119-128, 244-152.full_text 12539951

[B77] Payandeh J Pai EF Enzyme-driven speciation: crystallizing Archaea via lipid capture J Mol Evol 2007 64 364 374 10.1007/s00239-006-0141-8 17253090

[B78] Finn RD Tate J Mistry J Coggill PC Sammut SJ Hotz HR Ceric G Forslund K Eddy SR Sonnhammer EL Bateman A The Pfam protein families database Nucleic Acids Res 2008 36 D281 288 10.1093/nar/gkm960 18039703 PMC2238907

[B79] Sojka L Fucik V Krasny L Barvik I Jonak J YbxF, a protein associated with exponential-phase ribosomes in Bacillus subtilis J Bacteriol 2007 189 4809 4814 10.1128/JB.01786-06 17468242 PMC1913448

[B80] Stechmann A Cavalier-Smith T Evolutionary origins of Hsp90 chaperones and a deep paralogy in their bacterial ancestors J Eukaryot Microbiol 2004 51 364 373 10.1111/j.1550-7408.2004.tb00580.x 15218707

[B81] Cavalier-Smith T Origin of the cell nucleus, mitosis and sex: roles of intracellular coevolution Biol Direct 2010 5 7 10.1186/1745-6150-5-7 20132544 PMC2837639

[B82] van den Bosch H Schutgens RB Wanders RJ Tager JM Biochemistry of peroxisomes Annu Rev Biochem 1992 61 157 197 10.1146/annurev.bi.61.070192.001105 1353950

[B83] De Duve C Blueprint for a cell: the nature and origin of life 1991 Burlington, N.C.: N. Patterson

[B84] Cavalier-Smith T Nardon P, Gianinazzi-Pearson V, Grenier AM, Margulis L, Smith DC Symbiotic origin of peroxisomes Endocytobiology IV; Paris 1990 Institut national de la recherche agronomique 515 521

[B85] Gabaldon T Snel B van Zimmeren F Hemrika W Tabak H Huynen MA Origin and evolution of the peroxisomal proteome Biol Direct 2006 1 8 10.1186/1745-6150-1-8 16556314 PMC1472686

[B86] Schluter A Fourcade S Ripp R Mandel JL Poch O Pujol A The evolutionary origin of peroxisomes: an ER-peroxisome connection Mol Biol Evol 2006 23 838 845 10.1093/molbev/msj103 16452116

[B87] Gabaldon T Huynen MA Reconstruction of the proto-mitochondrial metabolism Science 2003 301 609 10.1126/science.1085463 12893934

[B88] Duhita N Le HA Satoshi S Kazuo H Daisuke M Takao S The origin of peroxisomes: The possibility of an actinobacterial symbiosis Gene 2009 450 18 24 10.1016/j.gene.2009.09.014 19818387

[B89] Race HL Herrmann RG Martin W Why have organelles retained genomes? Trends Genet 1999 15 364 370 10.1016/S0168-9525(99)01766-7 10461205

[B90] Allen JF Control of gene expression by redox potential and the requirement for chloroplast and mitochondrial genomes J Theor Biol 1993 165 609 631 10.1006/jtbi.1993.1210 8114509

[B91] Koonin EV Temporal order of evolution of DNA replication systems inferred by comparison of cellular and viral DNA polymerases Biol Direct 2006 1 39 10.1186/1745-6150-1-39 17176463 PMC1766352

[B92] Rothschild LJ Mancinelli RL Life in extreme environments Nature 2001 409 1092 1101 10.1038/35059215 11234023

[B93] Valentine DL Adaptations to energy stress dictate the ecology and evolution of the Archaea Nat Rev Microbiol 2007 5 316 323 10.1038/nrmicro1619 17334387

[B94] Fogg MJ Pearl LH Connolly BA Structural basis for uracil recognition by archaeal family B DNA polymerases Nat Struct Biol 2002 9 922 927 10.1038/nsb867 12415291

[B95] Isambert H Stein RR On the need for widespread horizontal gene transfers under genome size constraint Biol Direct 2009 4 28 10.1186/1745-6150-4-28 19703318 PMC2740843

[B96] Heddle JA Bielas JH Unifying concept of DNA repair: the polymerase scanning hypothesis Environ Mol Mutagen 2005 45 143 149 10.1002/em.20112 15672383

[B97] Bielas JH Non-transcribed strand repair revealed in quiescent cells Mutagenesis 2006 21 49 53 10.1093/mutage/gei073 16394029

[B98] Bell PJ The viral eukaryogenesis hypothesis: a key role for viruses in the emergence of eukaryotes from a prokaryotic world environment Ann N Y Acad Sci 2009 1178 91 105 10.1111/j.1749-6632.2009.04994.x 19845630

[B99] Bell PJ Viral eukaryogenesis: was the ancestor of the nucleus a complex DNA virus? J Mol Evol 2001 53 251 256 10.1007/s002390010215 11523012

[B100] Vermeij GJ Evolution and escalation : an ecological history of life 1987 Princeton, N.J.: Princeton University Press

[B101] Wishart DS Knox C Guo AC Cheng D Shrivastava S Tzur D Gautam B Hassanali M DrugBank: a knowledgebase for drugs, drug actions and drug targets Nucleic Acids Res 2008 36 D901 906 10.1093/nar/gkm958 18048412 PMC2238889

[B102] Beiko RG Harlow TJ Ragan MA Highways of gene sharing in prokaryotes Proc Natl Acad Sci USA 2005 102 14332 14337 10.1073/pnas.0504068102 16176988 PMC1242295

[B103] Sioud M Possot O Elie C Sibold L Forterre P Coumarin and quinolone action in archaebacteria: evidence for the presence of a DNA gyrase-like enzyme J Bacteriol 1988 170 946 953 2828337 10.1128/jb.170.2.946-953.1988 PMC210746

[B104] Jolly L Newell J Porcelli I Vincent SJ Stingele F Lactobacillus helveticus glycosyltransferases: from genes to carbohydrate synthesis Glycobiology 2002 12 319 327 10.1093/glycob/12.5.319 12070074

[B105] Hansen JL Moore PB Steitz TA Structures of five antibiotics bound at the peptidyl transferase center of the large ribosomal subunit J Mol Biol 2003 330 1061 1075 10.1016/S0022-2836(03)00668-5 12860128

[B106] Hansen JL Ippolito JA Ban N Nissen P Moore PB Steitz TA The structures of four macrolide antibiotics bound to the large ribosomal subunit Mol Cell 2002 10 117 128 10.1016/S1097-2765(02)00570-1 12150912

[B107] Pfister P Corti N Hobbie S Bruell C Zarivach R Yonath A Bottger EC 23S rRNA base pair 2057-2611 determines ketolide susceptibility and fitness cost of the macrolide resistance mutation 2058A-->G Proc Natl Acad Sci USA 2005 102 5180 5185 10.1073/pnas.0501598102 15795375 PMC555689

[B108] Vester B Douthwaite S Macrolide resistance conferred by base substitutions in 23S rRNA Antimicrob Agents Chemother 2001 45 1 12 10.1128/AAC.45.1.1-12.2001 11120937 PMC90232

[B109] Tu D Blaha G Moore PB Steitz TA Structures of MLSBK antibiotics bound to mutated large ribosomal subunits provide a structural explanation for resistance Cell 2005 121 257 270 10.1016/j.cell.2005.02.005 15851032

[B110] Gregory ST Cate JH Dahlberg AE Spontaneous erythromycin resistance mutation in a 23S rRNA gene, rrlA, of the extreme thermophile Thermus thermophilus IB-21 J Bacteriol 2001 183 4382 4385 10.1128/JB.183.14.4382-4385.2001 11418580 PMC95329

[B111] Cavalier-Smith T Predation and eukaryote cell origins: a coevolutionary perspective Int J Biochem Cell Biol 2009 41 307 322 10.1016/j.biocel.2008.10.002 18935970

[B112] Martin W Koonin EV Introns and the origin of nucleus-cytosol compartmentalization Nature 2006 440 41 45 10.1038/nature04531 16511485

[B113] Lopez-Garcia P Moreira D Selective forces for the origin of the eukaryotic nucleus Bioessays 2006 28 525 533 10.1002/bies.20413 16615090

[B114] Jekely G Origin of the nucleus and Ran-dependent transport to safeguard ribosome biogenesis in a chimeric cell Biol Direct 2008 3 31 10.1186/1745-6150-3-31 18652645 PMC2503971

[B115] Csuros M Miklos I Streamlining and large ancestral genomes in Archaea inferred with a phylogenetic birth-and-death model Mol Biol Evol 2009 26 2087 2095 10.1093/molbev/msp123 19570746 PMC2726834

[B116] Krokan H Wist E Krokan RH Aphidicolin inhibits DNA synthesis by DNA polymerase alpha and isolated nuclei by a similar mechanism Nucleic Acids Res 1981 9 4709 4719 10.1093/nar/9.18.4709 6795595 PMC327469

[B117] Forterre P Elie C Kohiyama M Aphidicolin inhibits growth and DNA synthesis in halophilic arachaebacteria J Bacteriol 1984 159 800 802 6204969 10.1128/jb.159.2.800-802.1984 PMC215722

[B118] Anbar AD Knoll AH Proterozoic ocean chemistry and evolution: a bioinorganic bridge? Science 2002 297 1137 1142 10.1126/science.1069651 12183619

[B119] Cavalier-Smith T Cell evolution and Earth history: stasis and revolution Philos Trans R Soc Lond B Biol Sci 2006 361 969 1006 10.1098/rstb.2006.1842 16754610 PMC1578732

[B120] Aravind L Iyer LM Koonin EV Comparative genomics and structural biology of the molecular innovations of eukaryotes Curr Opin Struct Biol 2006 16 409 419 10.1016/j.sbi.2006.04.006 16679012

[B121] Cavalier-Smith T Obcells as proto-organisms: membrane heredity, lithophosphorylation, and the origins of the genetic code, the first cells, and photosynthesis J Mol Evol 2001 53 555 595 10.1007/s002390010245 11675615

[B122] Blobel G Intracellular protein topogenesis Proc Natl Acad Sci USA 1980 77 1496 1500 10.1073/pnas.77.3.1496 6929499 PMC348522

[B123] Griffiths G Cell evolution and the problem of membrane topology Nat Rev Mol Cell Biol 2007 8 1018 1024 10.1038/nrm2287 17971839

[B124] Shindyalov IN Bourne PE Protein structure alignment by incremental combinatorial extension (CE) of the optimal path Protein Eng 1998 11 739 747 10.1093/protein/11.9.739 9796821

[B125] Edgar RC MUSCLE: multiple sequence alignment with high accuracy and high throughput Nucleic Acids Res 2004 32 1792 1797 10.1093/nar/gkh340 15034147 PMC390337

[B126] Waterhouse AM Procter JB Martin DM Clamp M Barton GJ Jalview Version 2--a multiple sequence alignment editor and analysis workbench Bioinformatics 2009 25 1189 1191 10.1093/bioinformatics/btp033 19151095 PMC2672624

[B127] Guindon S Delsuc F Dufayard JF Gascuel O Estimating maximum likelihood phylogenies with PhyML Methods Mol Biol 2009 537 113 137 full_text 19378142 10.1007/978-1-59745-251-9_6

[B128] Puigbo P Wolf YI Koonin EV Search for a 'Tree of Life' in the thicket of the phylogenetic forest J Biol 2009 8 59 10.1186/jbiol159 19594957 PMC2737373

[B129] Kelman Z White MF Archaeal DNA replication and repair Curr Opin Microbiol 2005 8 669 676 10.1016/j.mib.2005.10.001 16242991

[B130] Sarubbi E Monti F Corti E Miele A Selva E Mode of action of the microbial metabolite GE23077, a novel potent and selective inhibitor of bacterial RNA polymerase Eur J Biochem 2004 271 3146 3154 10.1111/j.1432-1033.2004.04244.x 15265034

[B131] Masullo M Cantiello P De Paola B Fiengo A Vitagliano L Zagari A Arcari P Valine 114 replacements in archaeal elongation factor 1 alpha enhanced its ability to interact with aminoacyl-tRNA and kirromycin Biochemistry 2002 41 14482 14488 10.1021/bi026428n 12463746

