# Supplementary figures and images for: The origin of a derived superkingdom: how a gram-positive bacterium crossed the desert to become an archaeon
Source: Biol Direct. 2011 Feb 28;6:16. doi: 10.1186/1745-6150-6-16 (PMC3056875; doi:10.1186/1745-6150-6-16)

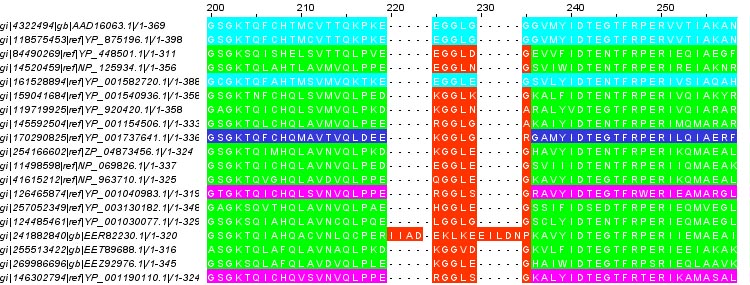

Supplement: Additional file 1 — Supplemental Figure 1. Alignment of RadA sequences from representative archaea. [file 1745-6150-6-16-S1.JPEG]

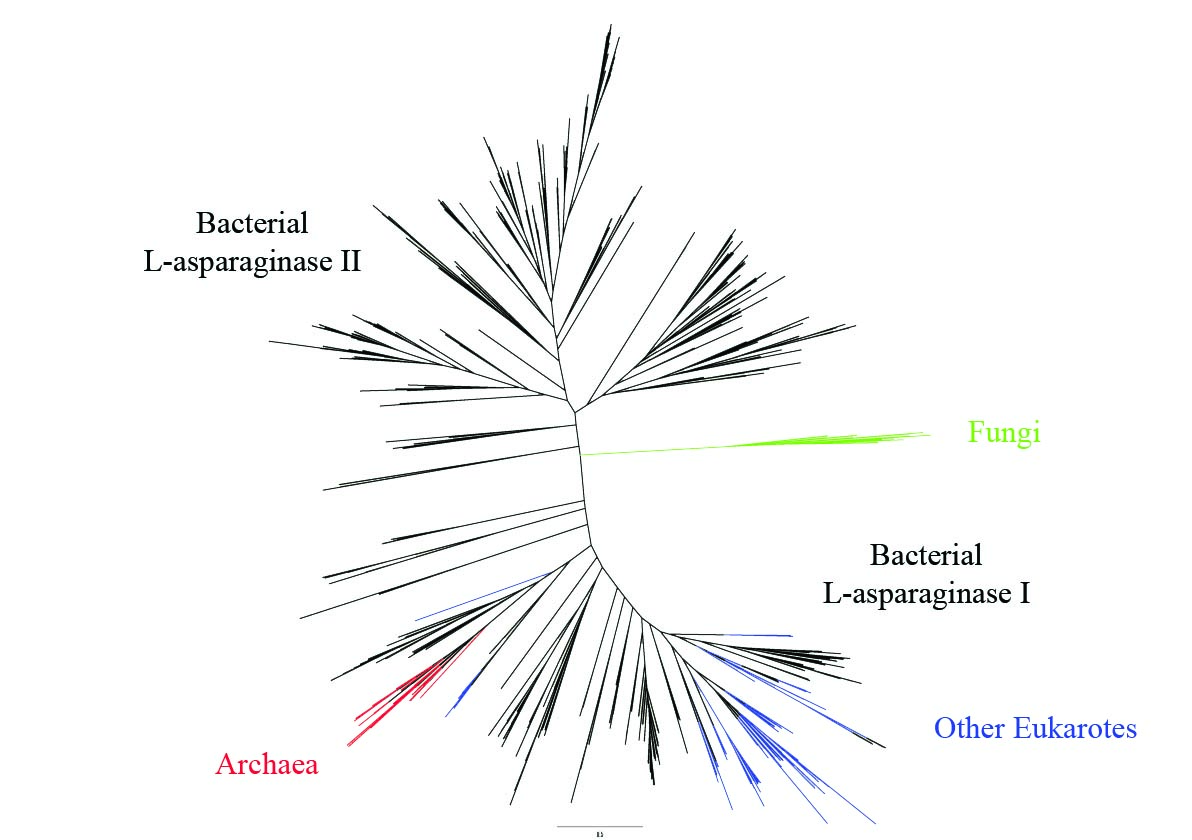

Supplement: Additional file 2 — Supplemental Figure 2. Maximum likelihood tree of GatD argues for multiple horizontal transfers. This tree is not well resolved, but it does not support archaeal ancestry for eukaryotic proteins. Euryarchaeal sequences are highlighted in green, crenarchaea are magenta, thaumarchaeota are cyan, and korarchaeota are blue. The region of the indel is highlighted in red. There is no informative indel in this gene as was initially reported. [file 1745-6150-6-16-S2.JPEG]

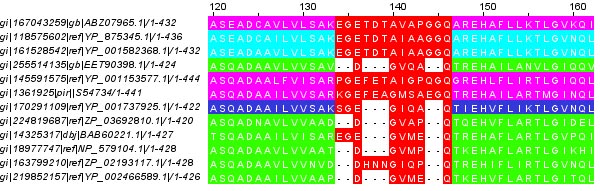

Supplement: Additional file 3 — Supplemental Figure 3. Sequence alignment of EF-1 from representative archaea. Euryarchaeal sequences are highlighted in green, crenarchaea are magenta, thaumarchaea are cyan, and korarchaeota are blue. The region of the indel is highlighted in red. This alignment implies several reversions. Therefore this indel is not robust enough to determine whether archaea are holophyletic or paraphyletic. [file 1745-6150-6-16-S3.JPEG]

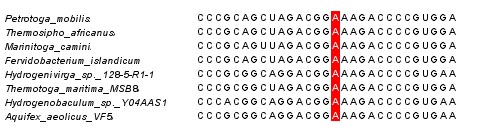

Supplement: Additional file 4 — Supplemental Figure 4. 23s rRNA A2058 (E. coli numbering) is well conserved across bacterial hyperthermophiles. This implies the conserved guanine in that position in archaea is not an adaptation to thermophily. [file 1745-6150-6-16-S4.JPEG]
